# Smokers Increasingly Motivated and Able to Quit as Smoking Prevalence Falls: Umbrella and Systematic Review of Evidence Relevant to the “Hardening Hypothesis,” Considering Transcendence of Manufactured Doubt

**DOI:** 10.1093/ntr/ntac055

**Published:** 2022-03-03

**Authors:** Miranda Harris, Melonie Martin, Amelia Yazidjoglou, Laura Ford, Robyn M Lucas, Eryn Newman, Emily Banks

**Affiliations:** National Centre for Epidemiology and Population Health, The Australian National University, Canberra, Australia; National Centre for Epidemiology and Population Health, The Australian National University, Canberra, Australia; National Centre for Epidemiology and Population Health, The Australian National University, Canberra, Australia; National Centre for Epidemiology and Population Health, The Australian National University, Canberra, Australia; National Centre for Epidemiology and Population Health, The Australian National University, Canberra, Australia; Research School of Psychology, College of Health and Medicine, The Australian National University, Canberra, Australia; National Centre for Epidemiology and Population Health, The Australian National University, Canberra, Australia

## Abstract

**Introduction:**

The “hardening hypothesis” proposes that as the prevalence of smoking in a population declines, there will be a “hardening” of the remaining smoker population. This review examines the evidence regarding smokers’ motivation, dependence, and quitting behavior as smoking prevalence declines, to assess whether population “hardening” (decreasing propensity to quit) or “softening” (the converse) is occurring.

**Methods:**

MEDLINE, PsychINFO, Scopus, Web of Science, and Cochrane Library were searched to July 2019, using terms related to smoking and hardening, for reviews and large, population-based repeat cross-sectional studies. There were additional searches of reference lists and citations of key research articles. Two reviewers screened half the titles and abstracts each, and two reviewers screened full texts independently using tested criteria. Four reviewers independently and systematically extracted data from eligible publications, with one reviewer per study, checked by another reviewer.

**Results:**

Of 265 titles identified, three reviews and ten repeat cross-sectional studies were included. Reviews concluded that hardening has not occurred among the general smoking population over time. Among repeated cross-sectional studies, five examined motivation, nine examined dependence, five examined hardcore smoking, and two examined quit outcomes. All but one study found a lack of hardening. Most found softening within the smoking population, consistent across hardening indicators, definitions, countries (and tobacco control environments), and time periods examined.

**Conclusions:**

Tobacco control reduces smoking prevalence and fosters a smoking population more amenable to evidence-based interventions. Based on the weight of the available evidence, the “hardening hypothesis” should be rejected and the reality of softening accepted.

**Implications:**

This umbrella review and systematic review provides a critical consideration of evidence from epidemiology and psychology and other fields regarding the “hardening hypothesis”—a persistent myth undermining tobacco control. It reaches the conclusion that the sum-total of the worldwide evidence indicates either “softening” of the smoking population, or a lack of hardening. Hence, tobacco control reduces smoking prevalence and fosters a smoking population more amenable to evidence-based interventions. The review indicates that the time has come to take active steps to combat the myth of hardening and to replace it with the reality of “softening.”

## Introduction

Tobacco use is a leading cause of preventable death and disability worldwide and there are international commitments to reducing smoking prevalence.^1^ In reducing prevalence, one critical factor is understanding the behavioral trajectory of the population of smokers who are yet to quit. The “hardening hypothesis” proposes that as the prevalence of smoking in a population declines, there will be a “hardening,” whereby smokers who are more resistant to established cessation interventions make up a greater proportion of the remaining smoker population.^2,3^ The hypothesis is based on the expressed concern that pressures to quit smoking from tobacco control policies and increasing social stigma of smoking could mean that smokers who found it relatively easy to quit would most readily cease smoking, and the smokers left behind would be increasingly resistant to tobacco control measures.^4^ The term “softening” has been coined to describe the opposite of hardening, whereby the smoking population displays behaviors characteristic of increasing willingness and/or ability to quit over time.

Indicators of hardening or softening can be categorized as measuring motivational or dependence hardening, proportion of hard-core smokers, and quit outcomes (Table 1).^5^ While the prevalence of socioeconomic disadvantage and of psychological distress among smokers have been postulated to be indicators of hardening, these are not direct measures of hardening.^6^

**Table 1.** Constructs and Indicators of Hardening Among Current Smokers

Hardening constructs	Indicators
Motivational hardening	Attitudes to smoking or tobacco control Quit attempts Quit intentions
Dependence hardening	Proportion of smokers who are daily smokers Proportion of smokers who are heavy smokers Mean number of cigarettes per day, among smokers Questionnaire measures of dependence (e.g. Nicotine Dependence Syndrome Scale)
Hard-core smoker	A composite of the indicators of motivational and dependence hardening above
Quit outcomes	Success on a given quit attempt or ability to remain abstinent on a given quit attempt Quit ratio (ratio of former smokers to ever smokers in a given population) Proportion of the eligible smoking population who have quit in a given time period

It should be noted that the hardening hypothesis is not a confirmed phenomenon and many tobacco control policies tend to produce softening as a necessary part of exerting their effects. For example, increasing costs of tobacco products, restrictions on places where people can smoke, graphic health warnings, and media campaigns all affect motivation and, along with support for cessation and reduced overall community prevalence of smoking, can improve quit outcomes.^7^ Reduction in the ability to smoke large numbers of cigarettes per day—including due to cost, restrictions on places where smoking is allowed, and lack of social acceptability—is likely to affect dependence.

One hypothetical concern is that previously effective population-level interventions could become less successful if a population hardens, requiring greater emphasis on individual-level cessation interventions to reach hardened smokers who would make up a greater proportion of the population of smokers.^3,5,8^ Evidence of hardening would also be considered to provide potential justification for increasing long-term nicotine replacement approaches—such as patches, gums, and e-cigarettes—as “harm reduction” for smokers who struggle to quit nicotine use.^2,4,9^

At the same time, it is important to note that the terms used in medical discourse are potentially powerful metaphors that may have consequences for the general public.^10,11^ A term like “hardening” may activate ideas like stability and resistance to change, and tends to imply that difficulty quitting is an irreversible quality of specific individuals or populations. There is a risk that continuing use of such terms could undermine smoking cessation and broader tobacco control by reinforcing detrimental and unsubstantiated beliefs. It is therefore important to review the current empirical evidence on whether hardening is actually occurring and, if it is not, what measures are necessary to avoid its continuing use, including as a justification for policy change.

The hardening hypothesis is most rigorously tested by examining changes in hardening or softening indicators within the population of smokers over extended periods of time, using a cohort or repeat cross-sectional study design.^5^ This review aims to summarize the contemporary evidence assessing the evidence for hardening or softening of the population of smokers and to consider strategies to align narratives with evidence.

## Methods

### Definitions of Hardening Constructs and Indicators

Motivational hardening may occur if the population of smokers become, on average, less motivated or willing to quit.^3,5^ Less motivated smokers are characterized by the absence of quit attempts or the lack of an intention to quit.^3^ A smoker’s attitude towards tobacco control measures has been proposed as an indirect measure of motivation to quit.^5^

Dependence hardening occurs if an increasing proportion of smokers are dependent (either physiologically dependent on nicotine or behaviorally on smoking).^3^ These smokers may experience multiple failed quit attempts and/or exhibit behavior consistent with high levels of dependence such as heavy consumption, smoking soon after waking (measured by time to first cigarette), and high scores on questionnaires measuring dependence.^3^ The average number of cigarettes smoked per day has been used to measure whether the average dependence of smokers is changing. Multiple unsuccessful quit attempts is also considered a marker of dependence.^5,12^

A hard-core smoker is usually conceptualized as a smoker who is highly unwilling and/or unable to quit and likely to remain this way.^3^ Although there is no agreed definition of a hard-core smoker, the categorization generally relates to both very low levels of motivation and very high levels of dependence.^13^ Common indicators used include nicotine dependence, regular smoking, lack of motivation or readiness to quit, and lack of recent quit attempts.^13^ Most definitions exclude smokers aged 25 years and younger, as these individuals are still establishing their smoking patterns.^13–15^ The concept of a hard-core smoker is an individual measure and is separate, but often related, to hardening, which is a population measure. It is possible to have hard-core smokers in a smoking population that does not show evidence of hardening over time. Conversely, the population of smokers may be hardening over time but the proportion of hard-core smokers may not change. These concepts are often linked in the published evidence in that the proportion of smokers who are classed as “hard-core” has been considered an indicator of hardening or softening of the smoking population.^3^

If hardening of the population of smokers were occurring due to reduced motivation or increased dependence, there would be a decline over time in the conversion of current smokers to former smokers.^5^ This is often measured by the “quit ratio”—the ratio of former smokers to ever smokers in a given population—or by the proportion of the eligible smoking population who have quit within the last twelve months.^3^ Success on a given quit attempt could also be considered a quit outcome.

### Literature Search, Screening, and Data Extraction

Reviews and primary research studies of repeat large population-based cross-sectional studies from Europe, UK, US, Canada, New Zealand, and Australia with measures of smoking outcomes ascertained at two or more time points, with a gap of at least 5 years between the first and last data point in the series, in line with another review of hardening,^12^ were identified through a combination of database searches and reference and citation searches. The work outlined in this paper was commissioned by the Australian Department of Health and primary research studies from Australia and countries with broadly comparable contexts, including achievements in tobacco control, were emphasized. MEDLINE, PsychINFO, Scopus, Web of Science, and Cochrane Library were searched up until July 2019 using a search strategy detailed in Supplementary Material 1.

Two review authors screened half the titles and abstracts each, independently. The two authors also screened all full texts using tested criteria, with disagreement about eligibility resolved through discussion involving a third reviewer. Studies were excluded if they were not representative of the general population or had less than 1000 participants for any survey year (full inclusion/exclusion criteria in Supplementary Material 2).

Four review authors independently extracted data from studies using piloted data extraction spreadsheets, with a check performed by another reviewer. The quality of included repeated cross-sectional studies was independently assessed by four review authors (two per study) using a tool adapted from the Joanna Briggs Institute Critical Appraisal Checklist for Studies Reporting Prevalence Data and the National Heart, Lung, and Blood Institute Study Quality Assessment Tool for Observational Cohort and Cross-Sectional Studies.^16,17^ As no systematic reviews were identified, the quality of the included reviews was not assessed. Disagreements were resolved through discussion between the two review authors, and through discussion involving a third reviewer when required. Author declarations of interest and other relevant information were reviewed and summarized. For interpretability, where relevant, this review reports on the change in the proportion of smokers meeting the hardening indicator definition over time.

## Results

**Figure 1.**
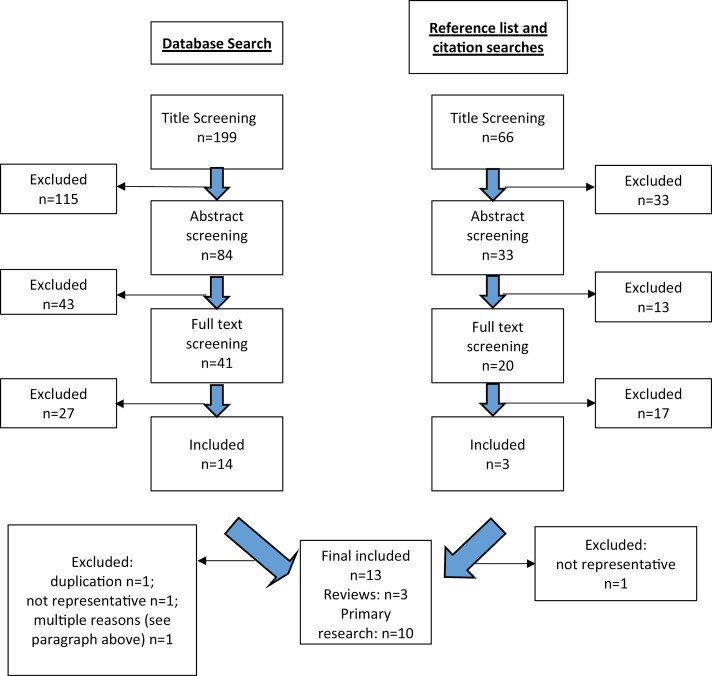
Flow diagram demonstrating study selection.

Of 265 titles identified, three reviews and ten repeat cross-sectional studies were identified for inclusion (Figure 1).

### Reviews

All three reviews conclude that hardening has not occurred among the general population of smokers, despite each considering different evidence.^3,6,12^ In 2003, Warner and Burns^3^ reviewed three empirical analyses on hardening and presented evidence against the case for hardening in the US population. They concluded that the proportion of hard-core smokers in the population was very small and, at the time of their review, there was little evidence that hardening was occurring at the population level.^3^ In 2011, Hughes^6^ updated the review by Warner and Burns^3^ and a review from the US Department of Health and Human Services,^18^ identifying two new studies on quit attempts, plotting quit ratios from the US National Health Interview Supplement, and reviewing five new studies relating to nicotine dependence. Hughes^6^ found no evidence of hardening among the general population of smokers, but did find evidence of hardening among treatment seekers. In 2019, Hughes^12^ undertook another review of 26 studies to assess whether there was a decrease over time in (1) conversion from current to former smoking; (2) quit attempts; or (3) success on a given quit attempt. None of the reviewed studies found evidence of hardening, and many found evidence of softening (Supplementary Material 3). Hughes (2019)^12^ provides the most recent and robust review of hardening, but does not include data on quit intentions, dependence, and attitudes on tobacco control.

### Primary Evidence

None of the ten repeated cross-sectional studies included had been considered in the reviews by Warner and Burns,^3^ Hughes (2011),^6^ or Hughes (2019).^12^ Although one of the studies included in Hughes (2019)^12^ has the same reference as Kulik and Glantz (2016)^19^ which is included in the current review, based on the information presented in Hughes (2019), it does not appear to be the same study. One study,^20^ which was excluded from the review of primary research, set out to replicate and critique the findings of another included study.^19^ Reasons for excluding this study were: duplication of data from the original study; adjustment for variables such as tobacco control policy that were highly correlated with smoking prevalence and likely to be mediators of softening over time; and potential competing interests of the authors.^20,21^

Of the ten repeated cross-sectional studies included, five examined motivation, nine examined measures of dependence, five examined hard-core smoking, and two examined quit outcomes over time. Eight studies examined hardening in the 2000s,^5,14,22-27^ and two examined hardening across both the 1990s and the 2000s.^19,28^ Four studies were conducted in the US,^19,25-27^ with one of these studies also examining 31 countries across Europe.^19^ The latter study^19^ presented analyses of European data for two hardening indicators; only one of these analyses has been reported in this review as the other analysis did not meet the minimum time period between data points required for inclusion. Two studies were conducted in Australia^23,24^ and there was one study each from New Zealand,^5^ Canada,^22^ Norway^28^ and England.^14^

An overview summary of the results is presented in Supplementary Material 4, with detailed findings presented in Supplementary Material 5. Supplementary Material 6 provides a summary of authors’ conflicts of interests.

### Motivation

International evidence from five studies on quit intentions and attempts indicates that, as smoking prevalence declines, the smoking population is either becoming more motivated to quit, or remaining stable in its motivation.^5,14,19,23,24^ For example, analysis of a series of large national household surveys in Australia shows the odds of having no plans to quit were significantly lower in 2010 compared with all previous years (odds ratio (OR) 0.87, 95% CI 0.77–0.98).^24^ The proportion of smokers in a subsequent state-based study in Victoria, Australia also shows that smokers were less likely over time to have no intention to quit, with those who had no intention of quitting in the next 30 days or the next six months decreasing between 2001 and 2016 (adjusted OR (aOR) per calendar year 0.95, 95% CI 0.93–0.96, *p*(trend) < .001, and aOR per calendar year 0.97, 95% CI 0.96–0.98, *p*(trend) < .001 respectively).^23^ The Victorian study also found that there was a significant decrease in the proportion of smokers who indicated they were happy to smoke for the rest of their lives (aOR per calendar year 0.97, 95% CI 0.95–0.99, *p*(trend) = .001).^23^ New Zealand smokers’ attitudes to tobacco control measures and goals, as a proxy measure for motivation, have softened over time or remained unchanged.^5^ Between 2008 and 2014, there was a steady increase over time in the proportion of daily smokers who supported banning smoking in all public places where children are likely to go (2008: 44.8%, 2014: 66.3%; aOR per two-year increment 1.16, 95% CI 1.08–1.25, p(trend) not reported).^5^ The proportion of daily smokers who agreed with reducing the number of places allowed to sell tobacco to make it less available showed no significant change, as did support for cigarettes and tobacco not being sold in New Zealand in ten years’ time.

### Dependence

Two studies in Australia,^23,24^ four in the US,^19,25-27^ and one in Canada,^22^ England,^14^ and New Zealand^5^ examined change in markers of dependence over time. The measures used to examine dependence differed across studies, with cigarettes per day being the most common measure and the proportion of either heavy smokers or daily smokers within the smoking population also frequently used. The definition of heavy smoking varied; some publications defined heavy smoking as at least 15 or 16 cigarettes per day while another used at least 25 cigarettes per day as the threshold. Other measures included time to first cigarette after waking and the Nicotine Dependence Syndrome Scale.

The available evidence indicates that dependence is on average declining or not changing in smokers, demonstrated by a decrease or no change in the proportion of smokers who were daily or heavy smokers,^5,23,25–27^ a decrease or no change in the proportion of smokers who were smoking soon after waking,^14,22,26^ no change in the proportion of smokers with four or more quit attempts of more than 24 hours in the past year^5^ and a decrease in dependence scores on the Nicotine Dependence Severity Scale.^27^ Of the smokers that continued to smoke, consumption, measured by average number of cigarettes per day, declined over time.^19^ For example, in Australia, the study conducted in the state of Victoria found that smokers were increasingly less likely to be daily or heavy smokers between 2001 and 2016 (among smokers, daily smoking 84.2% to 79.7%, aOR per calendar year 0.96, 95% CI 0.95–0.98; *p*(trend) < .001; heavy smoking 42.3% to 21.3%, aOR per calendar year 0.93, 95% CI 0.92–0.94),^23^ whilst the national study found no change between 2001 and 2010 (noting that no statistical test was reported for the dependence measure).^24^ The Victorian study found no variation in the change in prevalence of heavy smoking over time according to age, sex, education, or socioeconomic status.^23^ In their US study, Smith et al.^27^ examined sociodemographic factors and comorbidities, finding that declines in dependence severity (on the Nicotine Dependence Severity Scale) were greatest for smokers without any serious psychological distress. No significant variation in change in dependence severity over time was found according to sex, annual income, or age.^27^

### Hard-Core Smoking

Five studies examined hardening in the population of smokers over time based on data related to hard-core smoking.^14,22–24,28^ In Australia, there was no evidence of hardening as measured by change in proportion of the smoking population who were hard-core smokers. The Victorian study found a significant decline in the proportion of smokers who were hard-core between 2001 and 2016 (17.2% to 9.1% aOR per calendar year 0.94, 95% CI 0.92–0.96; *p*(trend) < .001),^23^ and the national study found no significant change over four waves from 2001 to 2010 (2001: 11.9%, 2004: 10.9%, 2007: 11.8%, 2010: 10.7% *p*(heterogeneity by wave) = .550).^24^

Brennan et al.^23^ undertook sensitivity analyses to explore the impact on the findings of using different definitions of hard-core smoker. In one definition, the criterion of not making a quit attempt within the past twelve months was replaced with having never attempted to quit. In two additional analyses, the heavy consumption criterion was removed as authors noted that cigarette consumption may be influenced by tobacco control policies, such as smoke-free policies, reducing the opportunities to smoke rather than reflecting the nicotine dependence of an individual. Regardless of the definition used, the proportion of smokers who were hard-core smokers decreased significantly over time, supporting the findings of the primary analysis.

Using nationally representative data from Australia, Clare et al.^24^ found that the change in the proportion of “hard-core” smokers over time varied according to socioeconomic status (*p*(interaction) = .025); between 2001 and 2010, the proportion of smokers being hard-core declined among people of higher socioeconomic status (2001: 9.3%, 2010: 6.7%) but remained static among those of lower socioeconomic status (2001: 13.7%, 2010: 13.7%). Victorian data^23^ also indicate a difference in changes in the proportion of hard-core smokers over time by level of education (*p*(interaction) < .017); however this was not significant at the authors’ prespecified *p* ≤ .01 level. The decline over time in the proportion of hard-core smokers (aOR per calendar year 0.97, 95% CI: 0.94-0.99, *p*(trend) = .012) was smaller in the group with lower education compared to that in the higher education group (aOR per calendar year 0.92, 95% CI: 0.90–0.94, *p*(trend) < .001). The proportion of hard-core smokers did not differ over time according to an area-based measure of socioeconomic status (*p*(interaction) = .434).

A Norwegian study using nationally representative data found evidence of softening, demonstrated by a decline in the proportion of smokers who were hard-core smokers over the period 1996 to 2009 (OR per increment in survey year (2 years) 0.90, 95% CI 0.88–0.93).^28^ There was no evidence of a change in the proportion of smokers who were hard-core in Canada between 2004 and 2010 using nationally representative data.^22^

An English study assessed data from two national datasets, both analyses finding there was an increase in the proportion of smokers who were defined as hard-core in England between 2000 and 2010 (UK General Lifestyle Survey *p*(trend) < .001; Health Survey for England *p*(trend) = .04).^14^ Based on graphs presented by the authors, the proportion of smokers who were hard-core was estimated to have increased by approximately 1% and 2% in the General Lifestyle Survey, and the Health Survey for England, respectively, over the eleven-year time period. However, when the two components of the hard-core smoker definition were examined separately, there was no statistically significant change over time in either survey in the odds of smokers who did not want to quit (*p*(trend) = .760 and .592 respectively), or for smokers who had their first cigarette within 30 minutes after waking (*p*(trend) = .288 and .785 respectively).

### Quitting Outcomes

One US study^19^ included an examination of the relationship between quit ratio and smoking prevalence and one New Zealand Study^5^ examined recent and sustained quit rates. The US study found that the quit ratio increased as smoking prevalence declined between 1992/93 and 2010/11: an increase of 1.13% (± 0.06 standard error, *p* < .001) for each 1% decrease in smoking prevalence.^19^ In the New Zealand study, authors found no significant change between 2008 and 2014 in recent quit rates (2008: 8.4%, 2014: 9.5%; aOR per two-year increment 1.03 (95% CI: 0.92–1.15)) or recent sustained quit rates (2008: 6.9%, 2014: 12.4%; aOR per two-year increment 1.12 (95% CI: 0.96–1.30)).^5^

## Discussion

Despite repeated concerns expressed regarding hardening, there is no evidence it is happening in smoking populations in the countries examined, with virtually all indicators consistent with softening or showing no significant change. The available evidence from studies from Australia, Canada, England, Europe, New Zealand, Norway, and the US does not indicate hardening of the population of smokers between 1992 and 2016, and in many cases shows softening—that is, becoming, on average, more motivated to quit and less dependent on smoking. The findings are consistent with the reviews by Warner and Burns,^3^ Hughes (2011)^12^ and Hughes (2019),^12^ which did not find evidence of hardening. They are also consistent with a recent review regarding the prevalence of hardcore smoking, published outside the timeframes of this review, incorporating publications to mid-2018.^29^ Based on the evidence to date, the lack of hardening within the population of smokers is almost completely consistent across the range of hardening indicators employed, their definitions, countries (and tobacco control environments), and time periods examined. Furthermore, the majority of smokers who have quit successfully since the 1960s have done so without any formal support, including heavy smokers.^2^

Hence, the current balance of evidence is against the occurrence of hardening, and a useful way forward is perhaps to consider that, given the scale and consistency of the evidence against it, it is unlikely that large amounts of supportive evidence will emerge in the near future.

Alongside clear evidence-based grounds to embrace “softening” as the reality of tobacco control, there are a range of other important considerations. The concept of the hard-core smoker perpetuates stigma and neither recognizes nor addresses appropriately the complex factors related to ongoing smoking.^13,30^ The continuing use of “hardening” metaphors risks reinforcing misleading and detrimental beliefs about factors that contribute to smoking cessation. Metaphors can shape the way people think about and view health hurdles, influencing their perceptions of personal control, how fatalistic they feel, and how challenging a given health goal, such as quitting smoking, seems to be.^11^ These linguistic devices can also be used by those with vested interests, including the tobacco industry, to influence public debate and policy outcomes.^31^ The concern here is that a given claim or idea—e.g., those who haven’t quit yet likely won’t, or need help via e-cigarettes—can accumulate credibility and belief via nonprobative evidence, such as how easy it is to imagine that scenario. Indeed, claims that evoke imagery tend to feel true, and once an idea like hardening has been repeated several times, such claims—whether they are true or false—tend to feel right.^32^ Even with knowledge that a claim like hardening came from the tobacco industry (a source that might be untrustworthy in this context), people often forget the source of a claim or fail to use it in their assessments of truth—so even in the face of a questionable source, simple repetition and high imagery tends to increase belief.^33,34^ The familiarity produced by the repetition of a claim or idea can also signal social consensus—that many others think so too,^35^ a psychological cue that guides how we think and how we act.

Having powerful metaphors and making them widely heard has the potential to shape public perceptions of health issues, public discourse, and what feels true, regardless of the current state of empirical evidence. Voltaire is paraphrased as saying that “Those who can make you believe absurdities can make you commit atrocities” ^36^ highlighting not only the importance of belief in governing action, but also the nature of power and influence, using misinformation. Portraying heavy addiction among smokers as an immutable property of the smoker themselves denies the active role the tobacco industry plays in creating and prolonging addiction and supports its calculated shift from perpetrator to “savior”—through investment in and promotion of products such as e-cigarettes and heated tobacco products—while continuing to profit from tobacco and nicotine products.^37^

More importantly, these data demonstrate a clear need to actively challenge and replace the hardening discourse with an evidence-based discourse about what truly happens as smoking prevalence falls. The greater the extent to which the scientific and public discourse centers around hardening, the more people may believe the hardening hypothesis.^32^ Even more worrying would be in the context of policy development, in which decisions around smoking interventions could be influenced by messages that felt intuitively appealing, rather than those with a foundation of robust empirical evidence. Such mistakes have been made in the past—for example, with tobacco smoking itself and with the intuitive appeal of “light” cigarettes—with devastating consequences that health systems around the world are still trying to address.^31^

Returning to metaphors and the power of language, when one is attempting to correct an unsupported or disputed claim, setting the rhetorical frame around a myth or unsubstantiated idea such as there is “no evidence for hardening,” without an attempt to carefully debunk it and provide alternative explanations, may actually increase the familiarity and perceived truth of the to-be-corrected content. Increasing evidence in cognitive psychology supports a different communication approach that makes the empirically supported fact the center of the discussion, making the core finding—in this case, evidence of softening—more memorable.^32,38^ Increasing evidence also suggests that warning people about classic disinformation tactics and refuting them in advance (e.g., the use of “Fake Experts” to sow doubt and distort perceived scientific consensus) can work to reduce vulnerability to misinformation.^39,40^ This “prebunking” teaches people about the “go-to” tactics of those intending to insert doubt or persuade and has been effective in helping people to identify when those tactics appear in information they encounter.^41^ The studies we have reviewed here suggest that such interventions may be warranted in the future if a hardening rhetoric continues, given that the large volume of evidence indicates shifting to softening is empirically valid and will lead to more evidence-based policy, practice, and public awareness.

Comprehensive and multifaceted tobacco control measures have proved effective in reducing the prevalence of smoking in many countries. These measures include smoke-free policies, mass media campaigns, plain packaging, graphic health warnings on packaging, price increases, and prohibitions on tobacco advertising, promotion, and sponsorship.^7,42^ Such measures actually act through softening: they make it more challenging for smokers to continue smoking large numbers of cigarettes by reducing opportunities to smoke, making it very expensive to do so, and reducing its social acceptability. These measures also increase smokers’ motivation to quit, including through awareness of harms.

In parallel with reductions in the general population, the prevalence of smoking has fallen in many groups with historically high prevalence, including Indigenous peoples.^43^ Where softening is occurring among the general population of smokers, it may be occurring to a varying extent within important subpopulations, such as smokers from low socioeconomic backgrounds and smokers experiencing psychological distress.^5^ While one study found that the odds of being a hard-core smoker in Australia declined over the study years to a greater extent among those from high compared to low socioeconomic status groups,^24^ another study found consistent softening patterns according to age, gender, socioeconomic status, and education.^23^ In disadvantaged populations, higher smoking prevalence relates to a range of interacting psychological, social, economic, and cultural factors.^7^ Furthermore, it is important that the myth of hardening is not confused with the clear evidence that smoking is more common among people of lower socioeconomic status. Irrespective of how smoking is characterized, tobacco control interventions should be equitable and aim to reduce smoking across all population groups.

This review focused on peer-reviewed published evidence designed specifically to address questions regarding the hardening hypothesis in the population of smokers. Repeat large population-based cross-sectional studies from high-income countries—with comprehensive tobacco control policies—with a gap of at least five years between the first and last data points in the series were included, with multiple authors independently extracting data. For certain hardening indicators, such as cigarettes per day and quit ratios, there are likely to be additional data in other publications, including government and technical reports. However, such publications often do not test statistically for change in indicators of hardening and may not be peer-reviewed.

The majority of primary research studies included in this review were of good quality. Most studies adjusted for relevant potential confounding factors over time, namely age and sex. Two studies adjusted for concomitant nicotine administration in the form of snus and nicotine replacement therapy^26,28^ which is relevant for the measurement of cigarettes per day if the use of concomitant nicotine-containing products has changed over time. A common limitation when assessing study quality was determining the validity of measures being used to assess hardening. Heterogeneity in the definitions and measurement of hardening indicators across studies makes it difficult to reliably ascertain the prevalence of hardened smoking in a population and to compare between studies and over time.^6,13,44^ This review examined patterns of hardening across a range of indicators in studies with data representing millions of people, and included high quality national and state-level representative population-based survey data spanning two decades. Despite the variability in the definitions of hard-core smoker observed in the primary research, sensitivity analyses support the findings of a lack of hardening regardless of the definition of hard-core smoker used. Unlike other recent reviews of the hardening hypothesis,^6,12^ the authors of this review do not have any competing interests.

In conclusion, in countries that have been successful in achieving relatively low smoking levels, declining smoking prevalence has been accompanied by increasing motivation to quit and reduced dependency among the smoking population—indicating softening or a lack of hardening over time. These findings provide a clear message of progress and improvement, highlighting the effectiveness of ongoing tobacco control measures in reducing the prevalence of smoking as well as increasing motivation to quit and reducing dependency among the population that continues to smoke. Based on the weight of the available evidence, the “hardening hypothesis” should not only be rejected—it should be replaced with active efforts to engage and educate the general public, researchers, policy makers, and practitioners about the genuine “softening” consequences of tobacco control.

## Supplementary Material

A Contributorship Form detailing each author’s specific involvement with this content, as well as any supplementary data, are available online at https://academic.oup.com/ntr.

ntac055_suppl_Supplementary_Material_S1

ntac055_suppl_Supplementary_Material_S2

ntac055_suppl_Supplementary_Material_S3

ntac055_suppl_Supplementary_Material_S4

ntac055_suppl_Supplementary_Material_S5

ntac055_suppl_Supplementary_Material_S6

ntac055_suppl_Supplementary_Taxonomy-form

## Data Availability

Not applicable – there are no new data associated with this article.

## References

[1] World Health Organization. WHO Framework Convention on Tobacco Control. Geneva, Switzerland: World Health Organization; 2003.

[2] Chapman S , Wakefield MA. Large-scale unassisted smoking cessation over 50 years: lessons from history for endgame planning in tobacco control. Tob Control. 2013;22(Suppl 1):i33–i35. 23591504 10.1136/tobaccocontrol-2012-050767 PMC3632984

[3] Warner KE , Burns DM. Hardening and the hard-core smoker: concepts, evidence, and implications. Nicotine Tob Res. 2003;5(1):37–48. 12745505 10.1080/1462220021000060428

[4] Chaiton MO , Cohen JE, Frank J. Population health and the hardcore smoker: Geoffrey Rose revisited. J Public Health Policy. 2008;29(3):307–318. 18701900 10.1057/jphp.2008.14

[5] Edwards R , Tu D, Newcombe R, Holland K, Walton D. Achieving the tobacco endgame: evidence on the hardening hypothesis from repeated cross-sectional studies in New Zealand 2008–2014. Tob Control. 2017;26(4):399–405. 27382047 10.1136/tobaccocontrol-2015-052860

[6] Hughes JR . The hardening hypothesis: is the ability to quit decreasing due to increasing nicotine dependence? A review and commentary. Drug Alcohol Depend. 2011;117(2-3):111–117. 21411244 10.1016/j.drugalcdep.2011.02.009 PMC3133840

[7] Greenhalgh EM , Scollo MM, Winstanley MH. Tobacco in Australia: Facts and issues. Cancer Council Victoria. www.TobaccoInAustralia.org.au Published 2020. Accessed.

[8] Burns DM , Warner KE. Smokers who have not quit: is cessation more difficult and should we change our strategies? In: National Cancer Institute, ed. Those Who Continue to Smoke. NIH Pub. No. 03-5370. Bethesda, MD: U.S. Department of Health and Human Services, Public Health Service, National Institutes of Health, National Cancer Institute 2003:11–31.

[9] Polosa R , Rodu B, Caponnetto P, Maglia M, Raciti CA. Fresh look at tobacco harm reduction: the case for the electronic cigarette. Harm Reduct J 2013;10(1):19. 24090432 10.1186/1477-7517-10-19 PMC3850892

[10] Scherer AM , Scherer LD, Fagerlin A. Getting ahead of illness: using metaphors to influence medical decision making. Med Decis Making. 2015;35:37–45. 24615273 10.1177/0272989X14522547

[11] Hauser D , Schwarz N. The war on prevention II: battle metaphors undermine cancer treatment and prevention and do not increase vigilance. Health Commun. 2020;35(13):1698–1704. 31496298 10.1080/10410236.2019.1663465

[12] Hughes JR . An update on hardening: a qualitative review. Nicotine Tob Res. 2019;22(6):867–871. 10.1093/ntr/ntz042 30868166

[13] Darville A , Hahn EJ. Hardcore smokers: what do we know? Addict Behav 2014;39(12):1706–1712. 25117846 10.1016/j.addbeh.2014.07.020

[14] Docherty G , McNeill A, Gartner C, Szatkowski L. Did hardening occur among smokers in England from 2000 to 2010? Addiction 2014;109(1):147–154. 24103060 10.1111/add.12359 PMC3933730

[15] Lam TH , Cheung YT, Leung DY, Abdullah AS, Chan SS. Effectiveness of smoking reduction intervention for hardcore smokers. Tob Induc Dis 2015;13(1):9. 25859176 10.1186/s12971-015-0034-y PMC4391680

[16] National Institute of Health—National Heart, Lung and Blood Institute. Quality Assessment Tool for Observational Cohort and Cross-Sectional Studies. https://www.nhlbi.nih.gov/health-topics/study-quality-assessment-tools. Accessed 10 May 2019.

[17] The Joanna Briggs Institute. The Joanna Briggs Institute Critical Appraisal tools for use in JBI Systematic Reviews: Checklist for Prevalence Studies. https://joannabriggs.org/research/critical-appraisal-tools.html. Published 2017. Accessed 10 May 2019.

[18] National Cancer Institute. Those Who Continue to Smoke. NIH Pub. No. 03-5370. Bethesda, MD: U.S. Department of Health and Human Services, National Institutes of Health, National Cancer Institute;2003.

[19] Kulik MC , Glantz SA. The smoking population in the USA and EU is softening not hardening. Tob Control. 2016;25(4):470–475. 26108654 10.1136/tobaccocontrol-2015-052329 PMC4690792

[20] Plurphanswat N , Rodu B. Is the smoking population in the United States really softening? Addiction 2016;111(7):1299–1303. 27177450 10.1111/add.13340

[21] Glantz S. Addiction refuses to allow discussion of industry ties to criticism of our “softening paper.” Center for Tobacco Control Research and Education. https://tobacco.ucsf.edu/addiction-refuses-allow-discussion-industry-ties-criticism-our-%E2%80%9Csoftening-paper%E2%80%9D. Published 2016. Accessed 21 January, 2020.

[22] Azagba S . Hardcore smoking among continuing smokers in Canada 2004-2012. Cancer Causes Control 2015;26(1):57–63. 25359304 10.1007/s10552-014-0482-3

[23] Brennan E , Greenhalgh EM, Durkin SJ, et al Hardening or softening? An observational study of changes to the prevalence of hardening indicators in Victoria, Australia, 2001–2016. Tob Control. 2019;29:252–257. 10.1136/tobaccocontrol-2019-054937 31147477

[24] Clare P , Bradford D, Courtney RJ, Martire K, Mattick RP. The relationship between socioeconomic status and “hardcore” smoking over time--greater accumulation of hardened smokers in low-SES than high-SES smokers. Tob Control. 2014;23(e2):e133–e138. 24706085 10.1136/tobaccocontrol-2013-051436

[25] Coady MH , Jasek J, Davis K, et al Changes in smoking prevalence and number of cigarettes smoked per day following the implementation of a comprehensive tobacco control plan in New York City. J Urban Health. 2012;89(5):802–808. 22544658 10.1007/s11524-012-9683-9 PMC3462825

[26] Goodwin RD , Wall MM, Gbedemah M, et al Trends in cigarette consumption and time to first cigarette on awakening from 2002 to 2015 in the USA: new insights into the ongoing tobacco epidemic. Tob Control. 2018;27(4):379–384. 28801362 10.1136/tobaccocontrol-2016-053601

[27] Smith PH , Rose JS, Mazure CM, Giovino GA, McKee SA. What is the evidence for hardening in the cigarette smoking population? Trends in nicotine dependence in the U.S., 2002–2012. Drug Alcohol Depend. 2014;142:333–340. 25064023 10.1016/j.drugalcdep.2014.07.003 PMC4158455

[28] Lund M , Lund KE, Kvaavik E. Hardcore smokers in Norway 1996–2009. Nicotine Tob Res. 2011;13(11):1132–1139. 21849413 10.1093/ntr/ntr166 PMC3203137

[29] Buchanan T , Magee C, See H, Kelly P. Tobacco harm reduction: are smokers becoming more hardcore? *J Public Health Pol*. 2020;41(1):286–302. 10.1057/s41271-020-00226-1 32366990

[30] West R , Jarvis MJ. Is “hardcore smoker” a useful term in tobacco control? Addiction 2018;113(1):3–4. 10.1111/add.14073 29226537

[31] Oreskes N , Conway EM. Defeating the merchants of doubt. Nature 2010;465:686–687. 20535183 10.1038/465686a

[32] Schwarz N , Newman E, Leach W. Making the truth stick and the myths fade: lessons from cognitive psychology. Behav Sci Policy. 2016;2(1):85–95.

[33] Henkel LA , Mattson ME. Reading is believing: the truth effect and source credibility. Conscious and Cogn. 2011;20:1705–1721. 10.1016/j.concog.2011.08.018 21978908

[34] Stanley ML , Yang BW, Marsh EJ. When the unlikely becomes likely: qualifying language does not influence later truth judgments. J Appl Res Mem Cogn 2019;8:118–129.

[35] Weaver K , Garcia SM, Schwarz N, Miller DT. Inferring the popularity of an opinion from its familiarity: a repetitive voice can sound like a chorus. J Pers Soc Psychol. 2007;92(5):821–833. 17484607 10.1037/0022-3514.92.5.821

[36] Voltaire. Collection des lettres sûr les miracles. Neufchâtel,1765.

[37] Tobacco Control Research Group. Tobacco Tactics: E-cigarettes. Department for Health, University of Bath. https://tobaccotactics.org/wiki/e-cigarettes/.Published2021.Updated15March2021. Accessed 5 May, 2021.

[38] Lewandowsky S , Cook J, Ecker UKH, et al. The Debunking Handbook 2020. Available at https://sks.to/db2020. Accessed Feb 2021.

[39] Blastland M , Freeman AL, van der Linden S, Marteau TM, Segehalter D. Five rules for evidence communication. Nature. 2020;587(7834):362–364. 33208954 10.1038/d41586-020-03189-1

[40] Cook J , Lewandowsky S, Ecker UKH. Neutralizing misinformation through inoculation: exposing misleading argumentation techniques reduces their influence. PLoS One. 2017;12(5):e0175799. 28475576 10.1371/journal.pone.0175799 PMC5419564

[41] Basol M , Roozenbeek J, van der Linden S. Good news about bad news: gamified inoculation boosts confidence and cognitive immunity against fake news. J Cogn 2020;3(2):1–9. 31934684 10.5334/joc.91 PMC6952868

[42] Intergovernmental Committee on Drugs. National Tobacco Strategy 2012-2018. Canberra: Commonwealth of Australia; 2012.

[43] Lovett R , Thurber K, Wright A, Maddox R, Banks E. Deadly progress: changes in Australian Aboriginal and Torres Strait Islander adult daily smoking, 2004–2015. Public Health Res Pract 2017;27(5):e2751742. 10.17061/phrp2751742 31044210

[44] Costa ML , Cohen JE, Chaiton MO, et al “Hardcore” definitions and their application to a population-based sample of smokers. Nicotine Tob Res. 2010;12(8):860–864. 20601409 10.1093/ntr/ntq103

