# Supplementary material for: Smokers Increasingly Motivated and Able to Quit as Smoking Prevalence Falls: Umbrella and Systematic Review of Evidence Relevant to the “Hardening Hypothesis,” Considering Transcendence of Manufactured Doubt
Source: Nicotine Tob Res. 2022 Mar 3;24(8):1321–8. doi: 10.1093/ntr/ntac055 (PMC9278822; doi:10.1093/ntr/ntac055)
Supplement: ntac055_suppl_Supplementary_Material_S1 [file ntac055_suppl_supplementary_material_s1.pdf]

Supplementary Material 1: Search strategy for the review of evidence for the 'hardening hypothesis'

MEDLINE, PyschINFO, Scopus, Web of Science and Cochrane Library were searched. The titles and abstracts were screened to isolate relevant publications. Full texts were then identified by two review authors and independently assessed against the selection criteria.

Any conflicts were discussed and if no consensus was reached the publication was reviewed by a third review author. Additional publications were identified via searches of reference lists and citations of the primary research articles and a recently published editorial on the hardening hypothesis. Data were systematically extracted from the publications using data extraction templates.

MEDLINE search terms:

1. Smoking/
2. Ex-Smokers/
3. Tobacco Smoking/
4. Smokers/
5. 1 or 2 or 3 or 4
6. hardened.mp.
7. hardening.mp.
8. "hard core".mp.
9. "hard-core".mp.
10. hardcore.mp.
11. "hardening hypothesis".mp.
12. softening.mp.
13. 6 or 7 or 8 or 9 or 10 or 11 or 12
14. 5 and 13

PsychINFO search terms:

1. smoking.mh.
2. ex-smokers.mh.
3. smokers.mh.
4. tobacco smoking.mh.
5. 1 or 2 or 3 or 4
6. hardened.mp.
7. hardening.mp.
8. "hard core".mp.
9. "hard-core".mp.
10. hardcore.mp.

11. "hardening hypothesis".mp.
12. softening.mp.
13. 6 or 7 or 8 or 9 or 10 or 11 or 12
14. 5 and 13 40

Scopus search terms:

1. TITLE-ABS-KEY (smoking OR smokers OR "ex-smokers" OR "tobacco smokers" AND hardening OR hardened OR "hard core" OR hardcore OR "hard-core" OR "hardening hypothesis" OR softening) AND DOCTYPE (ar OR re) AND (LIMIT-TO (SUBJAREA , "MEDI") OR LIMIT-TO (SUBJAREA , "SOCI") OR LIMIT-TO (SUBJAREA , "PSYC")) AND (LIMIT-TO (LANGUAGE , "English"))

Web of Science search terms:

1. (TS=(smoking OR smokers OR "ex-smokers" OR " tobacco smokers" AND hardening OR hardened OR "hard core" OR hardcore OR "hard-core" OR "hardening hypothesis" OR so_ening)) AND LANGUAGE: (English)

   Indexes=SCI-EXPANDED, SSCI, A&HCI, CPCI-S, CPCI-SSH, ESCI, CCR-EXPANDED, IC
   Timespan=All years

2. (TS=(smoking)) AND LANGUAGE: (English)

   Indexes=SCI-EXPANDED, SSCI, A&HCI, CPCI-S, CPCI-SSH, ESCI, CCR-EXPANDED, IC
   Timespan=All years

3. (TS=(smokers)) AND LANGUAGE: (English)

   Indexes=SCI-EXPANDED, SSCI, A&HCI, CPCI-S, CPCI-SSH, ESCI, CCR-EXPANDED, IC
   Timespan=All years

4. (TS=("ex-smokers")) AND LANGUAGE: (English)

   Indexes=SCI-EXPANDED, SSCI, A&HCI, CPCI-S, CPCI-SSH, ESCI, CCR-EXPANDED, IC
   Timespan=All years

5. (TS=("tobacco smoking")) AND LANGUAGE: (English)

   Indexes=SCI-EXPANDED, SSCI, A&HCI, CPCI-S, CPCI-SSH, ESCI, CCR-EXPANDED, IC
   Timespan=All years

6. #5 OR #4 OR #3 OR #2

   Indexes=SCI-EXPANDED, SSCI, A&HCI, CPCI-S, CPCI-SSH, ESCI, CCR-EXPANDED, IC
   Timespan=All years

7. (TS=(hardening)) AND LANGUAGE: (English)

   Indexes=SCI-EXPANDED, SSCI, A&HCI, CPCI-S, CPCI-SSH, ESCI, CCR-EXPANDED, IC
   Timespan=All years

8. (TS=(hardened)) AND LANGUAGE: (English)

   Indexes=SCI-EXPANDED, SSCI, A&HCI, CPCI-S, CPCI-SSH, ESCI, CCR-EXPANDED, IC Timespan=All years

9. (TS=(hardcore)) AND LANGUAGE: (English)

   Indexes=SCI-EXPANDED, SSCI, A&HCI, CPCI-S, CPCI-SSH, ESCI, CCR-EXPANDED, IC Timespan=All years

10. (TS=("hard-core")) AND LANGUAGE: (English)

    Indexes=SCI-EXPANDED, SSCI, A&HCI, CPCI-S, CPCI-SSH, ESCI, CCR-EXPANDED, IC Timespan=All years

11. (TS=("hard core")) AND LANGUAGE: (English)

    Indexes=SCI-EXPANDED, SSCI, A&HCI, CPCI-S, CPCI-SSH, ESCI, CCR-EXPANDED, IC Timespan=All years

12. (TS=("hardening hypothesis")) AND LANGUAGE: (English)

    Indexes=SCI-EXPANDED, SSCI, A&HCI, CPCI-S, CPCI-SSH, ESCI, CCR-EXPANDED, IC Timespan=All years

13. (TS=(so_ening)) AND LANGUAGE: (English)

    Indexes=SCI-EXPANDED, SSCI, A&HCI, CPCI-S, CPCI-SSH, ESCI, CCR-EXPANDED, IC Timespan=All years

14. #13 OR #12 OR #11 OR #10 OR #9 OR #8 OR #7

    Indexes=SCI-EXPANDED, SSCI, A&HCI, CPCI-S, CPCI-SSH, ESCI, CCR-EXPANDED, IC Timespan=All years

15. #14 AND #6

    Indexes=SCI-EXPANDED, SSCI, A&HCI, CPCI-S, CPCI-SSH, ESCI, CCR-EXPANDED, IC Timespan=All years

16. #14 AND #6

    Refined by: DOCUMENT TYPES: (ARTICLE OR REVIEW)

    Indexes=SCI-EXPANDED, SSCI, A&HCI, CPCI-S, CPCI-SSH, ESCI, CCR-EXPANDED, IC Timespan=All years

17. #14 AND #6

    Refined by: DOCUMENT TYPES: (ARTICLE OR REVIEW) AND DOCUMENT TYPES: (ARTICLE OR REVIEW)

Cochrane search terms:

1. MeSH descriptor: [Smokers] explode all trees
2. MeSH descriptor: [Ex-Smokers] explode all trees
3. MeSH descriptor: [Tobacco Smoking] explode all trees
4. MeSH descriptor: [Smoke] explode all trees

5. ("hard core"):ti,ab,kw
6. ("harden"):ti,ab,kw
7. ("hardening hypothesis"):ti,ab,kw
8. ("softening"):ti,ab,kw
9. ("hardcore"):ti,ab,kw
10. ("hard-core"):ti,ab,kw
11. (OR #1-#4)
12. (OR #5-#10)
13. #11 AND #12

The quality of the included studies was assessed independently by two reviewers, with discrepancies resolved by discussion and by adjudication of a third reviewer.
