# Supplementary material for: Smokers Increasingly Motivated and Able to Quit as Smoking Prevalence Falls: Umbrella and Systematic Review of Evidence Relevant to the “Hardening Hypothesis,” Considering Transcendence of Manufactured Doubt
Source: Nicotine Tob Res. 2022 Mar 3;24(8):1321–8. doi: 10.1093/ntr/ntac055 (PMC9278822; doi:10.1093/ntr/ntac055)
Supplement: ntac055_suppl_Supplementary_Material_S2 [file ntac055_suppl_supplementary_material_s2.pdf]

Supplementary Material 2: Inclusion and exclusion criteria

Inclusion criteria:

Study designs:	Systematic reviews, narrative reviews, population-based cohort studies, repeat cross-sectional studies of a representative sample of smokers.
Participants:	Adult tobacco smokers (as defined by study authors).
Outcome/ Condition:	Changes in hardening indicators (including proportion of smokers who are hard-core smokers) among smokers as defined by study authors, measured at two or more time points separated by at least five years.<br><br>Hardening indicators may include proportion of hard-core smokers, measure of motivation, dependence, and quit outcomes among the population of smokers.<br><br>Studies were also included if they assessed the hardening hypothesis or discussed their findings in the context of the hardening hypothesis.
Timing:	No restriction.
Setting:	Restricted to Europe, UK, US, Canada, New Zealand and Australia.
Language:	Articles reported in English.

Exclusion criteria:

Source:	Grey literature.
Study designs:	Anything other than a systematic review, narrative review, repeat cross-sectional study or population-based cohort study, such as intervention studies addressing pharmacological and non-pharmacological treatment of smoking cessation.
Participants:	Anyone other than adult tobacco smokers (as defined by study authors). Does not include general population of smokers, i.e., solely looks at a subgroup of smokers such as treatment seekers.
Outcome/ Condition:	Tobacco smoking not primary interest of the study e.g. other substance use is main outcome. Does not include at least one of the indicators of hardening or hard-core smoker.
Timing:	No exclusion criteria.
Setting:	Settings other than Europe, UK, US, Canada, New Zealand and Australia.
Language:	Articles not published in or translated into English.

Studies were also excluded if the survey they used was not representative of the general population or if the number of survey participants each year was less than 1000.
