# Supplementary material for: Smokers Increasingly Motivated and Able to Quit as Smoking Prevalence Falls: Umbrella and Systematic Review of Evidence Relevant to the “Hardening Hypothesis,” Considering Transcendence of Manufactured Doubt
Source: Nicotine Tob Res. 2022 Mar 3;24(8):1321–8. doi: 10.1093/ntr/ntac055 (PMC9278822; doi:10.1093/ntr/ntac055)
Supplement: ntac055_suppl_Supplementary_Material_S3 [file ntac055_suppl_supplementary_material_s3.pdf]

Supplementary Material 3: Summary table of the results of the studies included in Hughes (2019) review. Adapted from pp 2-3 of Hughes (2019).[1]

**Changes over time in conversion from current to former smoker**						
**Author**	**Survey years**	**Number of years covered**	**Number of data points**	**Data source**	**Location**	**Outcome**
Babb et al. (2017)[2]	2000-2015	15	4	NHIS	United States	Increase
Beard et al. (2019)[3]	1973-2016	44	44	APS, GLF, IHS	Great Britain	Increase[b]
Bosdriesz et al.[a] (2015a)[4]	1987-2012	20	12	DCSSH	Netherlands	Increase
Bosdriesz et al.[a] (2015)[5]	1988-2011	23	Multiple[b]	DCSSH	Netherlands	No change
Boyle et al. (2016)[6]	1999-2014	15	5	MATS	Minnesota	Increase
Burns et al. (2003)[7]	1992-1999	7	3	CTS	California	No change
Duval et al. (2008)[8]	1980-2002	20	5	MHS	United States	Increase
Fiore et al.[c] (1989)[9]	1974-1985	12	7	NHIS	United States	Increase[b]
ITC (2014)[10]	2002-2011	10	8	ITCS	United States	No change
King et al.[a] (2004)[11]	1990-2000	11	9	NHIS	United States	No change
Mendez et al.[a] (2016)[12]	1990-2014	24	4	NSDUH, NHIS	United States	Increase[b]
Messer et al.[a] (2007)[13]	1980-2000	20	20	CPS	United States	Increase[b]
Nagelhout et al.[a] (2012)[14]	2001-2008	8	8	DCSSH	Netherlands	No change
NCI (2010)[15]	1998-2008	10	10	NHIS	United States	No change
Pierce et al.[a] (1987)[16]	1974-1984	11	11	Gallup	Australia	Increase[b]
Pierce et al.[c] (1989)[17]	1974-1985	12	7	NHIS	United States	Increase[b]
Pierce et al. (2019)[18]	1974-2014	41	14	NHIS	United States	Increase[b]
Regidor et al.[a] (2015)[19]	1993-2012	20	8	SNHS	Spain	Increase
Szklo et al.[a] (2016)[20]	2008-2013	5	2	GATS	Brazil	Increase[b]
West et al. (2017)[21]	2007-2017	11	11	STS	England	Increase
**Median (IQR)[d]**	**1990-2001**	**13.5 (10.25-20)**	**8 (5-11)**			

APS=Annual Population Survey, CPS=Current Population Survey, CTS=California Tobacco Survey, DCSSH=Dutch Continuous Survey of Smoking Habits, GATS=Global Adult Tobacco Survey, GLF=General Lifestyle Survey, IHS=Integrated Household Survey, IQR=Interquartile range, ITCS=International Tobacco Control Survey, MATS=Minnesota Adult Tobacco Survey, MHS=Minnesota Heart Survey, NHIS=National Health Interview Survey, NSDUH=National Survey on Drug Use and Health, SNHS=Spanish National Health Survey, STS=Smoking Toolkit Survey

a Adjusted for covariates or subgroup analysis.

b Statistically significant.

c Data for Fiore et al. and Pierce et al. from same dataset and time period.

d IQR= Interquartile Range, that is, 25th and 75th percentile.

Changes over time in quit attempts						
**Author**	**Survey years**	**Number of years covered**	**Number of data points**	**Data source**	**Location**	**Outcome**
Babb et al. (2017)[2]	2000-2015	15	4	NHIS	United States	Increase
Gitchell et al. (2016)[22]	2009-2014	6	6	NHIS	United States	No change
ITC (2014)[10]	2002-2011	10	8	ITCS	United States	Increase
Kulik and Glantz[a] (2016)[23]	1997-2015	19	19	NHIS	United States	Increase[b]
Kulik and Glantz (2019)[24]	1990-2008	19	8	CTS	California	Increase[b]
Lavinghouze et al. (2015)[25]	2001-2013	13	13	BRFSS	United States	Increase[b]
Malacher et al.[a] (2011)[26]	2001-2010	10	10	NHIS	United States	Increase
West et al. (2017)[21]	2007-2017	11	11	STS	England	No change
**Median (IQR)c**	**2001-2013.5**	**12 (10,17)**	**9 (7,12)**			
**Success of a given quit attempt**						
**Author**	**Survey years**	**Number of years covered**	**Number of data points**	**Data source**	**Location**	**Outcome**
Beard et al. (2016)[27]	2006-2015	10	120	STS	England	Increase[b]
Brown and West[a] (2017)[28]	2007-2017	10	10	STS	England	Increase[b]
Gitchell et al. (2016)[22]	2009-2014	5	6	NHIS	United States	No change
Reid et al. (2014)[29]	2004-2013	9	13	CTADS	Canada	No change
West et al. (2017)[21]	2007-2017	11	11	STS	England	Increase
**Median (IQR)c**	**2007-2015**	**10 (7,10.5)**	**11 (8,66.5)**			

BRFSS=Behavioural Risk Factor Surveillance System, CTADS=Canadian Tobacco, Alcohol and Drugs Survey, CTS=California Tobacco Survey, IQR=Interquartile range, ITCS=International Tobacco Control Survey, NHIS=National Health Interview Survey, STS=Smoking Toolkit Survey

a Adjusted for covariates or subgroup analysis.

b Statistically significant.

c IQR, that is, 25th and 75th percentile.

References

1. Hughes JR. An Update on Hardening: A Qualitative Review. *Nicotine Tob Res* 2019; **22**(6): 867-71.
2. Babb S. Quitting smoking among adults—United States, 2000–2015. *MMWR Morb Mortal Wkly Rep* 2017; **65**.
3. Beard EV, West R, Jarvis M, Michie S, Brown J. 'S'-shaped curve: modelling trends in smoking prevalence, uptake and cessation in Great Britain from 1973 to 2016. *Thorax* 2019; **74**(9): 875-81.
4. Bosdriesz JR, Willemsen MC, Stronks K, Kunst AE. Socioeconomic inequalities in smoking cessation in 11 European countries from 1987 to 2012. *J Epidemiol Community Health* 2015; **69**(9): 886-92.
5. Bosdriesz JR, Nagelhout GE, Stronks K, Willemsen MC, Kunst AE. The association between tobacco control policy and educational inequalities in smoking cessation in the Netherlands from 1988 through 2011. *Nicotine Tob Res* 2015; **17**(11): 1369-76.
6. Boyle R OGE, D'Silva J. Are current Minnesota smokers more nicotine addicted? Testing the hardening hypothesis, 1999-2014. 2016. www.srnt.org/page/past. (accessed 19 December, 2018).
7. Burns DM, Major JM, Anderson CM, Vaughn JW. Changes in cross-sectional measures of cessation, numbers of cigarettes smoked per day, and time to first cigarette-California and national data. Those Who Continue to Smoke: Is Achieving Abstinence Harder and Do We Need to Change Our Interventions Smoking and Tobacco Control Monograph 15: U.S. Department of Health and Human Services, Public Health Service, National Institutes of Health, National Cancer Institute; 2003: 101-25.
8. Duval S, Jacobs DR, Barber C, et al. Trends in cigarette smoking: the Minnesota Heart Survey, 1980–1982 through 2000–2002. *Nicotine Tob Res* 2008; **10**(5): 827-32.
9. Fiore MC, Novotny TE, Pierce JP, Hatziandreu EJ, Patel KM, Davis RM. Trends in cigarette smoking in the United States: the changing influence of gender and race. *JAMA* 1989; **261**(1): 49-55.
10. Project ITC. ITC United States National Report: Findings from Wave 1 to 8 surveys (2002-2011). Waterloo, Ontario, Canada: University of Waterloo and Charleston, SC: Medical University of South Carolina, 2014.
11. King G, Polednak A, Bendel RB, Vilsaint MC, Nahata SB. Disparities in smoking cessation between African Americans and Whites: 1990–2000. *Am J Public Health* 2004; **94**(11): 1965-71.
12. Méndez D, Tam J, Giovino GA, Tsodikov A, Warner KE. Has smoking cessation increased? An examination of the US adult smoking cessation rate 1990–2014. *Nicotine Tob Res* 2016; **19**(12): 1418-24.
13. Messer K, Pierce JP, Zhu S-H, et al. The California Tobacco Control Program's effect on adult smokers:(1) Smoking cessation. *Tob Control* 2007; **16**(2): 85-90.
14. Nagelhout GE, de Korte-de Boer D, Kunst AE, et al. Trends in socioeconomic inequalities in smoking prevalence, consumption, initiation, and cessation between 2001 and 2008 in the Netherlands. Findings from a national population survey. *BMC public health* 2012; **12**(1): 303.
15. National Cancer Institute. Cancer trends progress report-2009/2010 update. 2010. http://progressreport.cancer.gov. (accessed April 16, 2010).
16. Pierce JP, Aldrich RN, Hanratty S, Dwyer T, Hill D. Uptake and quitting smoking trends in Australia 1974–1984. *Prev Med* 1987; **16**(2): 252-60.
17. Pierce JP, Fiore MC, Novotny TE, Hatziandreu EJ, Davis RM. Trends in cigarette smoking in the United States: educational differences are increasing. *JAMA* 1989; **261**(1): 56-60.
18. Pierce JP, Shi Y, McMenamin SB, et al. Trends in lung cancer and cigarette smoking: California compared to the rest of the United States. *Cancer Prevention Research* 2019; **12**(1): 3-12.
19. Regidor E, Pascual C, Giráldez-García C, Galindo S, Martínez D, Kunst AE. Impact of tobacco prices and smoke-free policy on smoking cessation, by gender and educational group: Spain, 1993–2012. *International Journal of Drug Policy* 2015; **26**(12): 1215-21.
20. Szklo AS, de Souza MC, Szklo M, de Almeida LM. Smokers in Brazil: who are they? *Tob Control* 2016; **25**(5): 564-70.

21. West R, Brown J. Latest trends on smoking in England from the smoking toolkit study. 2017. www.smokinginengland.info (accessed 19 December, 2018).
22. Gitchell JG, Shiffman S, Sembower MA. Trends in serious quit attempts in the United States, 2009–14. *Addiction* 2017; **112**(5): 897-900.
23. Kulik MC, Glantz SA. The smoking population in the USA and EU is softening not hardening. *Tob Control* 2016; **25**(4): 470-5.
24. Kulik MC, Glantz SA. Similar softening across different racial and ethnic groups of smokers in California as smoking prevalence declined. *Prev Med* 2019; **120**: 144-9.
25. Lavinghouze SR, Malarcher A, Jama A, Neff L, Debrot K, Whalen L. Trends in quit attempts among adult cigarette smokers—United States, 2001–2013. *MMWR Morb Mortal Wkly Rep* 2015; **64**(40): 1129-35.
26. Centers for Disease Control Prevention. Quitting smoking among adults--United States, 2001-2010. *MMWR Morb Mortal Wkly Rep* 2011; **60**(44): 1513.
27. Beard E, West R, Michie S, Brown J. Association between electronic cigarette use and changes in quit attempts, success of quit attempts, use of smoking cessation pharmacotherapy, and use of stop smoking services in England: time series analysis of population trends. *BMJ* 2016; **354**: i4645.
28. Brown J, West R. Quit success rates in England 2007-2017. *Smoking in Britain* 2017; **5**: 1-8.
29. Reid JL, Hammond D, Rynard FL, Madill CL, R B. Tobacco Use in Canada: Patterns and Trends, 2017 Edition. Waterloo, ON: Propel Centre for Population Health Impact, University of Waterloo.
