# Supplementary material for: Smokers Increasingly Motivated and Able to Quit as Smoking Prevalence Falls: Umbrella and Systematic Review of Evidence Relevant to the “Hardening Hypothesis,” Considering Transcendence of Manufactured Doubt
Source: Nicotine Tob Res. 2022 Mar 3;24(8):1321–8. doi: 10.1093/ntr/ntac055 (PMC9278822; doi:10.1093/ntr/ntac055)
Supplement: ntac055_suppl_Supplementary_Material_S4 [file ntac055_suppl_supplementary_material_s4.pdf]

Supplementary Material 4: Overview of the results from 10 repeated cross-sectional studies in Australia and similar countries examining hardening indicators

Authors	Indicator	Finding
Motivation – Quit intention		
**Australia**		
Clare et al (2014)[1]	No plan to quit	Softening
Brennan et al (2019)[2]	No intention to quit in the next 30 days	Softening
Brennan et al (2019)[2]	No intention to quit in the next 6 months	Softening
Brennan et al (2019)[2]	Happy to smoke for the rest of their lives	Softening
**International**		
Docherty et al (2014)[3]	Low motivation to quit	Neither hardening nor softening
Motivation – Quit attempts		
**Australia**		
Clare et al (2014)[1]	No quit attempt in the past 12 months	No statistical test reported (the authors reported that the proportion of smokers with no quit attempt in the past 12 months was consistent across the four waves)
Brennan et al (2019)[2]	No quit attempt in the past 12 months	Softening
Brennan et al (2019)[2]	No quit attempt in the past 5 years	Softening
Brennan et al (2019)[2]	Never attempted to quit	Neither hardening nor softening
**International**		
Kulik and Glantz (2016)[4]	Made a quit attempt in the past 12 months (US)	Softening
Kulik and Glantz (2016)[4]	Made a quit attempt in the past 12 months (EU)	Neither hardening nor softening
Edwards et al (2017)[5]	No quit attempts in the past 12 months	Neither hardening nor softening
Motivation – Attitudes to tobacco control		
**International**		
Edwards et al (2017)[5]	Agree with banning smoking in all public places where children are likely to go	Softening

Edwards et al (2017)[5]	Agree the number of places allowed to sell cigarettes and tobacco should be reduced	Neither hardening nor softening
Edwards et al (2017)[5]	Supported cigarettes and tobacco should not be sold in New Zealand in 10 years' time	Neither hardening nor softening
Dependence		
**Australia**		
Clare et al (2014)[1]	Heavy smoking	No statistical test reported (the authors reported that the proportion of heavy smokers was consistent across the four waves)
Brennan et al (2019)[2]	Daily smoking	Softening
Brennan et al (2019)[2]	Heavy smoking	Softening
**International**		
Coady et al (2012)[6]	Cigarettes per day in current smokers	Softening
Coady et al (2012)[6]	Heavy daily smoking	Softening
Docherty et al (2014)[3]	Time to first cigarette ≤ 30 minutes after waking	Neither hardening nor softening
Docherty et al (2014)[3]	Time to first cigarette ≥ 30 minutes after waking	Neither hardening nor softening
Smith et al (2014)[7]	Heavy smoking	No statistical test reported
Smith et al (2014)[7]	Nicotine Dependence Syndrome Scale scores	Softening
Azagba (2015)[8]	Time to first cigarette ≤ 5 minutes and/or ≤ 30 minutes after waking	Neither hardening nor softening
Kulik and Glantz (2016)[4]	Cigarettes per day in current smokers	Softening
Edwards et al (2017)[5]	Daily smoking	Neither hardening nor softening
Edwards et al (2017)[5]	Daily smoking with 4 or more quit attempts in the past 12 months	Neither hardening nor softening
Goodwin et al (2018)[9]	Heavy smoking	Softening
Goodwin et al (2018)[9]	Time to first cigarette < 30 minutes after waking	Softening

Hard-core smokers		
**Australia**		
Clare et al (2014)[1]	Hard-core smokers	Neither hardening nor softening
Brennan et al (2019)[2]	Hard-core smokers	Softening
Brennan et al (2019)[2]	Given up giving up	No statistical test conducted
**International**		
Lund et al (2011)[10]	Daily heavy smoking	Softening
Docherty et al (2014)[3]	Hard-core smokers	Hardening
Azagba (2015)[8]	Hard-core smokers	Neither hardening nor softening
Quit outcomes		
**International**		
Kulik and Glantz (2016)[4]	Quit ratio	Softening
Edwards et al (2017)[5]	Recent quit rate	Neither hardening nor softening
Edwards et al (2017)[5]	Recent sustained quit rate	Neither hardening nor softening

References

1. Clare P, Bradford D, Courtney RJ, Martire K, Mattick RP. The relationship between socioeconomic status and 'hardcore' smoking over time--greater accumulation of hardened smokers in low-SES than high-SES smokers. *Tob Control* 2014; **23**(e2): e133-8.
2. Brennan E, Greenhalgh EM, Durkin SJ, Scollo MM, Hayes L, Wakefield MA. Hardening or softening? An observational study of changes to the prevalence of hardening indicators in Victoria, Australia, 2001-2016. *Tob Control* 2019; **29**: 252-7.
3. Docherty G, McNeill A, Gartner C, Szatkowski L. Did hardening occur among smokers in England from 2000 to 2010? *Addiction* 2014; **109**(1): 147-54.
4. Kulik MC, Glantz SA. The smoking population in the USA and EU is softening not hardening. *Tob Control* 2016; **25**(4): 470-5.
5. Edwards R, Tu D, Newcombe R, Holland K, Walton D. Achieving the tobacco endgame: evidence on the hardening hypothesis from repeated cross-sectional studies in New Zealand 2008-2014. *Tob Control* 2017; **26**(4): 399-405.
6. Coady MH, Jasek J, Davis K, Kerker B, Kilgore EA, Perl SB. Changes in smoking prevalence and number of cigarettes smoked per day following the implementation of a comprehensive tobacco control plan in New York City. *J Urban Health* 2012; **89**(5): 802-8.
7. Smith PH, Rose JS, Mazure CM, Giovino GA, McKee SA. What is the evidence for hardening in the cigarette smoking population? Trends in nicotine dependence in the U.S., 2002-2012. *Drug Alcohol Depend* 2014; **142**: 333-40.
8. Azagba S. Hardcore smoking among continuing smokers in Canada 2004-2012. *Cancer Causes Control* 2015; **26**(1): 57-63.
9. Goodwin RD, Wall MM, Gbedemah M, et al. Trends in cigarette consumption and time to first cigarette on awakening from 2002 to 2015 in the USA: new insights into the ongoing tobacco epidemic. *Tob Control* 2018; **27**(4): 379-84.
10. Lund M, Lund KE, Kvaavik E. Hardcore smokers in Norway 1996-2009. *Nicotine Tob Res* 2011; **13**(11): 1132-9.
