# Supplementary material for: Smokers Increasingly Motivated and Able to Quit as Smoking Prevalence Falls: Umbrella and Systematic Review of Evidence Relevant to the “Hardening Hypothesis,” Considering Transcendence of Manufactured Doubt
Source: Nicotine Tob Res. 2022 Mar 3;24(8):1321–8. doi: 10.1093/ntr/ntac055 (PMC9278822; doi:10.1093/ntr/ntac055)
Supplement: ntac055_suppl_Supplementary_Material_S5 [file ntac055_suppl_supplementary_material_s5.pdf]

## Supplementary Material 5: Review of primary research studies. Detailed findings

| **AUSTRALIA: Motivation – quit intentions, quit attempts, no quit intentions, no quit attempts** | | | | | | |
|---|---|---|---|---|---|---|
| **Publication details (author, publication year, survey years, number of years covered, number of data points, location, data source)** | **Smoking prevalence in total population** | **Measure** | **Key findings in total population** | **Key findings in smoking population** | **Hardening, softening or no change** | **Quality assessment** |
| **Clare et al (2014)**[1]<br><br>2001 to 2010; 10; 4<br><br>Australia<br><br>National Drug Strategy Household Survey | 2001: 22.4%<br>2010: 19.0% | <u>Quit Intention</u><br>Proportion of current smokers with no plan to quit<br><br>Current smokers: daily, weekly or less than weekly; aged ≥ 18 years<br><br>*Smokers were asked whether they intend to quit.* | <u>Absolute Prevalence (%):</u><br>2001: 7.4%[~]<br>2010: 6.0%[~] | <u>Proportion (%):</u><br>2001: 33.2%<br>2004: 34.4%<br>2007: 35.1%,<br>2010: 31.4%<br><br>There was a significant association between wave and having no plan to quit (p = 0.004), with the odds of not having plans to quit significantly lower in 2010 compared with previous years (OR = 0.87; 95% CI: 0.77 to 0.98; p = 0.026).<br><br>*Adjusted for age, sex, SEIFA, education, and other sociodemographic variables.* | **Softening** evidenced by a statistically significant decrease in the odds of smokers not having plans to quit | Good |

| **AUSTRALIA: Motivation – quit intentions, quit attempts, no quit intentions, no quit attempts** | | | | | | |
|---|---|---|---|---|---|---|
| **Publication details (author, publication year, survey years, number of years covered, number of data points, location, data source)** | **Smoking prevalence in total population** | **Measure** | **Key findings in total population** | **Key findings in smoking population** | **Hardening, softening or no change** | **Quality assessment** |
| **Clare et al (2014)**[1]<br><br>2001 to 2010; 10; 4<br><br>Australia<br><br>National Drug Strategy Household Survey | 2001: 22.4%<br>2010: 19.0% | <u>Quit Attempt</u><br>Proportion of current smokers with no quit attempt in the past 12 months<br><br>Current smokers: daily, weekly or less than weekly; aged ≥ 18 years<br><br>*Smokers were asked whether they had made a quit attempt in the past 12 months.* | <u>Absolute Prevalence (%):</u><br>2001: 12.2%˜<br>2010: 10.7%˜ | <u>Proportion (%):</u><br>2001: 54.3%<br>2004: 57.1%<br>2007: 55.5%<br>2010: 56.4%<br><br>The authors reported that the proportion of smokers who reported no quit attempt in past 12 months was consistent across the four waves. Statistical significance not reported.<br><br>*Adjusted for age, sex, SEIFA, education, and other sociodemographic variables.* | **No statistical test reported** | Good |

| AUSTRALIA: Motivation – quit intentions, quit attempts, no quit intentions, no quit attempts | | | | | | |
|---|---|---|---|---|---|---|
| **Publication details (author, publication year, survey years, number of years covered, number of data points, location, data source)** | **Smoking prevalence in total population** | **Measure** | **Key findings in total population** | **Key findings in smoking population** | **Hardening, softening or no change** | **Quality assessment** |
| **Brennan et al (2019)[2]**<br><br>2001-2016; 16; 16<br><br>Victoria, Australia<br><br>The Victorian Smoking and Health Survey | | Quit Intention<br>Proportion of current smokers with no intention to quit in the next 30 days<br><br>Current smokers: smoked any tobacco, daily, weekly or less than weekly; aged ≥ 26 years<br><br>*Smokers who indicated they were considering quitting in the next 6 months*<br>*Q. 'Are you seriously considering quitting smoking in the next 6 months'*<br>*A. 'Yes', participants were then asked whether they planned to quit within the next 30 days.* | Absolute Prevalence (%):<br>2001: 16.9%~<br>2016: 9.4%~ | Proportion (%):<br>2001: 83.9%<br>2002: 86.3%<br>2003: 84.5%<br>2004: 86.3%<br>2005: 84.0%<br>2006: 83.7%<br>2007: 80.7%<br>2008: 79.3%<br>2009: 84.0%<br>2010: 83.4%<br>2011: 78.0%<br>2012: 77.4%<br>2013: 75.8%<br>2014: 77.7%<br>2015: 73.4%<br>2016: 72.1%<br><br>Linear Trend<br>Adjusted odds ratio (aOR) = 0.95 (95% CI: 0.93-0.96) $p < 0.001$<br><br>*Adjusted for sex, age, education and socioeconomic status* | **Softening** evidenced by a statistically significant decrease in the proportion of smokers who had no intention to quit in the next 30 days | Good |

| AUSTRALIA: Motivation – quit intentions, quit attempts, no quit intentions, no quit attempts | | | | | | |
|---|---|---|---|---|---|---|
| **Publication details (author, publication year, survey years, number of years covered, number of data points, location, data source)** | **Smoking prevalence in total population** | **Measure** | **Key findings in total population** | **Key findings in smoking population** | **Hardening, softening or no change** | **Quality assessment** |
| **Brennan et al (2019)[2]**<br><br>2001-2016; 16; 16<br><br>Victoria, Australia<br><br>The Victorian Smoking and Health Survey | 2001: 20.1%<br>2016: 13.0%<br><br>aOR = 0.98 (95% CI: 0.98 to 0.99)<br><br>(≥ 26 years of age) | Quit Intention<br>Proportion of current smokers who had no intention to quit in the next 6 months<br><br>Current smokers: smoked any tobacco, daily, weekly or less than weekly; aged ≥ 26 years<br><br>*Q. 'Are you seriously considering quitting smoking in the next 6 months?'* | Absolute Prevalence (%):<br>2001: 9.2%[+]<br>2016: 5.1%[+] | Proportion (%):<br>2001: 45.6%<br>2002: 49.9%<br>2003: 45.1%<br>2004: 47.6%<br>2005: 46.9%<br>2006: 46.5%<br>2007: 39.9%<br>2008: 38.1%<br>2009: 40.6%<br>2010: 39.6%<br>2011: 36.0%<br>2012: 36.6%<br>2013: 36.5%<br>2014: 40.5%<br>2015: 36.4%<br>2016: 38.9%<br><br>Linear Trend<br>aOR = 0.97 (95% CI: 0.96-0.98)<br>$p < 0.001$<br><br>*Adjusted for sex, age, education and socioeconomic stats* | **Softening** evidenced by a statistically significant decrease in the proportion of smokers who had no intention to quit in the next 6 months | Good |

| AUSTRALIA: Motivation – quit intentions, quit attempts, no quit intentions, no quit attempts | | | | | | |
|---|---|---|---|---|---|---|
| **Publication details (author, publication year, survey years, number of years covered, number of data points, location, data source)** | **Smoking prevalence in total population** | **Measure** | **Key findings in total population** | **Key findings in smoking population** | **Hardening, softening or no change** | **Quality assessment** |
| **Brennan et al (2019)[2]**<br><br>2001-2016; 16; 16<br><br>Victoria, Australia<br><br>The Victorian Smoking and Health Survey | | Happy to smoke<br>Proportion of current smokers who were happy to smoke for the rest of their lives<br><br>Current smokers: smoked any tobacco, daily, weekly or less than weekly; aged ≧ 26 years<br><br>*Q. 'Do you think you should quit sometime in the future, or are you happy to smoke for the rest of your life?'* | Absolute Prevalence (%):<br>2001: 3.8%~<br>2016: 1.6%~ | Proportion (%):<br>2001: 18.9%<br>2002: 20.1%<br>2003: 16.9%<br>2004: 14.0%<br>2005: 10.3%<br>2006: 11.7%<br>2007: N/A<br>2008: N/A<br>2009: N/A<br>2010: 11.8%<br>2011: 11.2%<br>2012: N/A<br>2013: 9.2%<br>2014: 12.3%<br>2015: 13.5%<br>2016: 12.0%<br><br>Linear Trend<br>aOR = 0.97 (95% CI: 0.95-0.99)<br>p = 0.001<br><br>*Adjusted for sex, age, education and socioeconomic status* | **Softening** evidenced by a statistically significant decrease in the proportion of smokers who were happy to smoke for the rest of their lives | Good |

| AUSTRALIA: Motivation – quit intentions, quit attempts, no quit intentions, no quit attempts | | | | | | |
|---|---|---|---|---|---|---|
| **Publication details (author, publication year, survey years, number of years covered, number of data points, location, data source)** | **Smoking prevalence in total population** | **Measure** | **Key findings in total population** | **Key findings in smoking population** | **Hardening, softening or no change** | **Quality assessment** |
| **Brennan et al (2019)[2]**<br><br>2001-2016; 16; 16<br><br>Victoria, Australia<br><br>The Victorian Smoking and Health Survey | | Quit Attempt<br>Proportion of current smokers who had made no quit attempt in the past 12 months<br><br>Current smokers: smoked any tobacco, daily, weekly or less than weekly; aged ≥ 26 years | Absolute Prevalence (%):<br>2001: 12.5%˜<br>2016: 7.1%˜ | Proportion (%):<br>2001: 62.3%<br>2002: 61.2%<br>2003: 64.3%<br>2004: 63.8%<br>2005: 69.5%<br>2006: 67.0%<br>2007: 65.1%<br>2008: 61.7%<br>2009: 64.5%<br>2010: 63.6%<br>2011: 59.7%<br>2012: 64.7%<br>2013: 59.8%<br>2014: 57.6%<br>2015: 62.1%<br>2016: 54.4%<br><br>Linear trend:<br>aOR = 0.98 (95% CI: 0.97-0.99)<br>p = 0.001<br><br>*Adjusted for sex, age, education and socioeconomic status* | **Softening** evidenced by a statistically significant decrease in the proportion of smokers who had made no quit attempt in the past 12 months | Good |

| AUSTRALIA: Motivation – quit intentions, quit attempts, no quit intentions, no quit attempts | | | | | | |
|---|---|---|---|---|---|---|
| **Publication details (author, publication year, survey years, number of years covered, number of data points, location, data source)** | **Smoking prevalence in total population** | **Measure** | **Key findings in total population** | **Key findings in smoking population** | **Hardening, softening or no change** | **Quality assessment** |
| **Brennan et al (2019)[2]**<br><br>2001-2016; 16; 16<br><br>Victoria, Australia<br><br>The Victorian Smoking and Health Survey | | <u>Quit Attempt</u><br>Proportion of current smokers who had made no quit attempt in the past 5 years<br><br>Current smokers: smoked any tobacco, daily, weekly or less than weekly; aged ≥ 26 years | <u>Absolute Prevalence (%):</u><br>2001: 7.3%˜<br>2016: 3.4%˜ | <u>Proportion (%):</u><br>2001: 36.2%<br>2002: 35.9%<br>2003: 33.4%<br>2004: 31.7%<br>2005: 34.3%<br>2006: 29.4%<br>2007: 31.9%<br>2008: 32.6%<br>2009: 30.4%<br>2010: 32.1%<br>2011: 25.1%<br>2012: 30.9%<br>2013: 24.3%<br>2014: 28.8%<br>2015: 29.7%<br>2016: 25.8%<br><br><u>Linear Trend</u><br>aOR = 0.97 (95% CI: 0.96-0.99)<br>$p < 0.001$<br><br>*Adjusted for sex, age, education and socioeconomic status* | **Softening** evidenced by a statistically significant decrease in the proportion of smokers who had made no quit attempt in the past 5 years | Good |

**AUSTRALIA: Motivation – quit intentions, quit attempts, no quit intentions, no quit attempts**

| Publication details (author, publication year, survey years, number of years covered, number of data points, location, data source) | Smoking prevalence in total population | Measure | Key findings in total population | Key findings in smoking population | Hardening, softening or no change | Quality assessment |
|---|---|---|---|---|---|---|
| **Brennan et al (2019)**[2]<br><br>2001-2016; 16; 16<br><br>Victoria, Australia<br><br>The Victorian Smoking and Health Survey | 2001: 20.1%<br>2016: 13.0%<br><br>aOR = 0.98 (95% CI: 0.98 to 0.99)<br><br>(≧ 26 years of age) | <u>Quit Attempts</u><br>Proportion of current smokers who had never attempted to quit<br><br>Current smokers: smoked any tobacco, daily, weekly or less than weekly; aged ≥ 26 years,<br><br>*Q. 'Approximately how many times, if any, have you tried to give up smoking?'*<br>*Q. 'How long ago did your last attempt to quit smoking end?'* | <u>Absolute Prevalence (%):</u><br>2001: 3.9%~<br>2016: 2.0%~ | <u>Proportion (%):</u><br>2001: 19.6%<br>2002: 22.5%<br>2003: 18.8%<br>2004: 18.5%<br>2005: 17.5%<br>2006: 15.4%<br>2007: 17.0%<br>2008: 16.9%<br>2009: 17.1%<br>2010: 18.0%<br>2011: 12.5%<br>2012: 17.8%<br>2013: 15.3%<br>2014: 17.2%<br>2015: 18.9%<br>2016: 15.0%<br><br><u>Linear Trend:</u><br>aOR = 0.98 (95% CI: 0.97-1.00)<br>p = 0.05<br><br>*Adjusted for sex, age, education and socioeconomic status* | **Neither hardening nor softening** evidenced by no significant change in the proportion of smokers who had never attempted to quit (as per the authors' pre-specified significance level of p ≤ 0.01) | Good |

| INTERNATIONAL-Motivation – quit intentions, quit attempts, no quit intentions, no quit attempts | | | | | | |
|---|---|---|---|---|---|---|
| **Publication details (author, publication year, survey years, number of years covered, number of data points, location, data source)** | **Smoking prevalence in total population** | **Measure** | **Key findings in total population** | **Key findings in smoking population** | **Hardening, softening or no change** | **Quality assessment** |
| **Docherty et al (2014)[3]**<br><br>2000-2010; 11; 11<br><br>England<br><br>General Lifestyle Survey | 2000: 26%<br>2010: 20%<br>(≥ 16 years of age) | Low motivation to quit<br>Proportion of current smokers ≥ 26 years of age, who do not want to quit<br><br>*Q. 'Would you like to give up smoking altogether?'* | Not reported, unable to calculate | Adjusted OR (95% CI):<br>2000: 1.00 Ref<br>2001: 1.04 (0.92–1.17)<br>2002: 0.96 (0.85–1.09)<br>2003: 1.04 (0.93–1.17)<br>2004: 0.92 (0.82–1.05)<br>2005: 0.91 (0.81–1.02)<br>2006: 0.87 (0.77–0.98)<br>2007: 0.93 (0.81–1.06)<br>2008: 1.06 (0.93–1.21)<br>2009: 1.04 (0.91–1.19)<br>2010: 1.01 (0.88–1.16)<br>P(trend) = 0.760<br><br>Prevalence (estimated):<br>2000: ~29%$^{+}$<br>2010: ~27%$^{+}$<br><br>*Adjusted for age, sex and socioeconomic status* | **Neither hardening nor softening** evidenced by no significant trend in the proportion of current smokers who had low motivation to quit | Good |

| INTERNATIONAL-Motivation – quit intentions, quit attempts, no quit intentions, no quit attempts | | | | | | |
|---|---|---|---|---|---|---|
| **Publication details (author, publication year, survey years, number of years covered, number of data points, location, data source)** | **Smoking prevalence in total population** | **Measure** | **Key findings in total population** | **Key findings in smoking population** | **Hardening, softening or no change** | **Quality assessment** |
| **Docherty et al (2014)[3]**<br><br>2000-2010; 11; 11<br><br>England<br><br>Health Survey for England | 2000: 26%[#]<br>2010: 20%[#]<br>(≥ 16 years of age) | Low motivation to quit<br>Proportion of current smokers ≥ 26 years of age, who do not want to quit<br><br>*Q. 'Would you like to give up smoking altogether?'* | Not reported, unable to calculate | Adjusted OR (95% CI):<br>2000: 1.00 Ref<br>2001: 1.01 (0.89-1.15)<br>2002: 1.14 (0.98-1.32)<br>2003: 0.98 (0.86-1.12)<br>2004: 0.95 (0.81-1.11)<br>2005: 0.90 (0.78-1.05)<br>2006: 1.01 (0.89-1.16)<br>2007: 0.95 (0.81-1.11)<br>2008: 1.13 (0.99-1.29)<br>2009: 1.13 (0.95-1.35)<br>2010: 0.82 (0.65-1.03)<br>p(trend) = 0.592<br><br>Prevalence (estimated):<br>2000: ~31%[+]<br>2010: ~33%[+]<br><br>*Adjusted for age, sex and socioeconomic status* | **Neither hardening nor softening** evidenced by no significant trend in the proportion of current smokers who reported 'no' to the question 'would you like to give up smoking altogether?' | Good |
| **Kulik and Glantz (2016)[4]**<br><br>1992/1993 – 2011/2012; 18; 348 (7 waves)<br><br>Note: not all states had data available for all years.<br><br>US<br><br>Tobacco Use Supplement of the Current Population Survey (TUS-CPS) | Not reported, unable to calculate | Quit Attempt<br>Proportion of everyday smokers, ≥ 18 years of age, who had stopped smoking for 1 day or longer because they were trying to quit smoking during the past 12 months<br><br>*Q. 'During the past 12 months, have you stopped smoking for one day or longer because you were trying to quit smoking?'* | Not reported, unable to calculate | A 1% decline in smoking prevalence is associated with a 0.55% (±0.07 SE, $p < 0.001$) increase in quit attempt prevalence | **Softening** as evidenced by a statistically significant increase in quit attempts | Fair |

| INTERNATIONAL-Motivation – quit intentions, quit attempts, no quit intentions, no quit attempts | | | | | | |
|---|---|---|---|---|---|---|
| **Publication details (author, publication year, survey years, number of years covered, number of data points, location, data source)** | **Smoking prevalence in total population** | **Measure** | **Key findings in total population** | **Key findings in smoking population** | **Hardening, softening or no change** | **Quality assessment** |
| **Kulik and Glantz (2016)**[4]<br><br>2006-2012; 6; 87 (3 waves)<br><br>Note: not all countries had data for all years<br><br>European data for 31 countries<br><br>Eurobarometer (EB) survey between 2006 and 2012 | 2006: 32% smokers<br>2012: 28% current smokers | Quit Attempt<br>Proportion of current smokers who had made a quit attempt in the previous 12 months<br><br>Current smokers: smoked at least 100 cigarettes in lifetime and currently smoking every day or for some days; ≥ 18 years of age | Not reported, unable to calculate | A 1% decline in smoking prevalence is associated with a 0.09% (±0.014 SE, $p = 0.53$) increase in quit attempts | **Neither hardening nor softening** evidenced by no significant association between smoking prevalence and quit attempts | Fair |

| INTERNATIONAL-Motivation – quit intentions, quit attempts, no quit intentions, no quit attempts | | | | | | |
|---|---|---|---|---|---|---|
| **Publication details (author, publication year, survey years, number of years covered, number of data points, location, data source)** | **Smoking prevalence in total population** | **Measure** | **Key findings in total population** | **Key findings in smoking population** | **Hardening, softening or no change** | **Quality assessment** |
| **Edwards et al (2017)[5]**<br><br>2008-2014; 7; 4<br><br>New Zealand<br><br>The Health and Lifestyle Survey (HLS) | <u>Daily Smoking Prevalence (%) (95% CI)</u><br>2008: 16.9 (14.3-19.5)<br>2010: 15.8 (12.8-18.8)<br>2012: 14.6 12.9-16.4<br>2014: 14.4 12.4-16.4<br><br>Difference 2014 vs 2008 (95% CI):<br>-2.5% (-5.8 to 0.8) | <u>Quit Attempts</u><br>Proportion of daily smokers, ≥ 15 years of age, who had no quit attempts of 24 hours or longer in the previous year | <u>Absolute Prevalence (%):</u><br>2008: 9.3%~<br>2014: 8.5%~ | <u>Proportion (%) (95% CI):</u><br>2008: 54.9 (46.7 to 63.1)<br>2010: 49.4 (39.8 to 59.0)<br>2012: 54.8 (48.3 to 61.2)<br>2014: 59.2 (52.6 to 65.8)<br><br>diff 2014 vs 2008: 4.3 (-6.2 to 1.5)<br><br><u>Adjusted OR (95% CI):</u><br>2008: 1.0 Ref<br>2010: 0.81 (0.48 to 1.36)<br>2012: 1.00 (0.66 to 1.52)<br>2014: 1.19 (0.78 to 1.83)<br><br><u>Linear Trend</u><br>aOR = 1.04 (95% CI: 0.97 to 1.11)<br><br>*Adjusted for age, gender and ethnicity*<br><br>Findings were similar for current smokers (smoke at least once a month) but data not shown. | **Neither hardening nor softening** evidenced by no significant change in the proportion of daily smokers who reported making no quit attempts of 24 hours or longer in the previous year | Good |

| INTERNATIONAL-Motivation – quit intentions, quit attempts, no quit intentions, no quit attempts | | | | | | |
|---|---|---|---|---|---|---|
| **Publication details (author, publication year, survey years, number of years covered, number of data points, location, data source)** | **Smoking prevalence in total population** | **Measure** | **Key findings in total population** | **Key findings in smoking population** | **Hardening, softening or no change** | **Quality assessment** |
| **Edwards et al (2017)[5]**<br><br>2008-2014; 7; 4<br><br>New Zealand<br><br>The Health and Lifestyle Survey (HLS) | | Attitudes to tobacco control<br>Proportion of daily smokers, ≥ 15 years of age, who agreed with banning smoking in all public places where children are likely to go | Absolute Prevalence (%):<br>2008: 7.6%~<br>2014: 9.6%~ | Proportion (%) (95% CI):<br>2008: 44.8 (36.3-53.3)<br>2010: 51.8 (41.4-62.1)<br>2012: 58.6 (52.3-64.9)<br>2014: 66.3 (59.1-73.4)<br>Diff 2014 vs 2008: 21.5 (10.4-32.6)<br><br>Adjusted OR (95% CI)<br>2008: 1<br>2010: 1.31 (0.76-2.24)<br>2012: 1.73 (1.13-2.64)<br>2014: 2.45 (1.53-3.93)<br><br>Linear Trend:<br>aOR = 1.16 (95% CI: 1.08-1.25)<br><br>*Adjusted for age, gender and ethnicity*<br><br>No mention of findings for current smokers. | **Softening** evidenced by a statistically significant increase in the proportion of daily smokers who agreed with banning smoking in all public places where children go | Good |

| INTERNATIONAL-Motivation – quit intentions, quit attempts, no quit intentions, no quit attempts | | | | | | |
|---|---|---|---|---|---|---|
| **Publication details (author, publication year, survey years, number of years covered, number of data points, location, data source)** | **Smoking prevalence in total population** | **Measure** | **Key findings in total population** | **Key findings in smoking population** | **Hardening, softening or no change** | **Quality assessment** |
| **Edwards et al (2017)[5]**<br><br>2008-2014; 7; 4<br><br>New Zealand<br><br>The Health and Lifestyle Survey (HLS) | | Attitudes to tobacco control<br>The proportion of daily smokers, ≥ 15 years of age, who agreed the number of places allowed to sell cigarettes and tobacco should be reduced to make them less easily available | Absolute Prevalence (%):<br>2008: 5.51%~<br>2014: 5.07%~ | Proportion (%) (95% CI):<br>2008: 32.6 (25.4 to 39.8)<br>2010: 32.3 (24.6 to 39.9)<br>2012: 42.4 (35.7 to 49.1)<br>2014: 35.2 (28.5 to 41.8)<br><br>Diff 2014 vs 2008: 2.6 (-7.2 to 12.3)<br><br>Adjusted OR (95% CI)<br>2008: 1<br>2010: 0.98 (0.61 to 1.59)<br>2012: 1.51 (0.99 to 2.31)<br>2014: 1.12 (0.72 to 1.74)<br><br>Linear Trend:<br>aOR = 1.04 (95% CI: 0.97 to 1.11)<br><br>*Adjusted for age, gender and ethnicity*<br><br>No mention of findings for current smokers. | **Neither hardening nor softening** as evidenced by no significant change in the proportion of daily smokers who agreed the number of places allowed to sell cigarettes and tobacco should be reduced to make them less easily available | Good |

| INTERNATIONAL-Motivation – quit intentions, quit attempts, no quit intentions, no quit attempts | | | | | | |
|---|---|---|---|---|---|---|
| **Publication details (author, publication year, survey years, number of years covered, number of data points, location, data source)** | **Smoking prevalence in total population** | **Measure** | **Key findings in total population** | **Key findings in smoking population** | **Hardening, softening or no change** | **Quality assessment** |
| **Edwards et al (2017)[5]**<br><br>2008-2014; 7; 4<br><br>New Zealand<br><br>The Health and Lifestyle Survey (HLS) | | Attitudes to tobacco control<br>The proportion of daily smokers, ≥ 15 years of age, who supported an 'endgame' type goal – cigarettes and tobacco should not be sold in New Zealand in 10 years' time | Absolute Prevalence (%):<br>2008: 4.39%~<br>2014: 3.96%~ | Proportion (%) (95% CI):<br>2008: 26% (18.9-33.1)<br>2010: 19.8% (14.2-25.4)<br>2012: 32.6% (26.4-38.9)<br>2014: 27.5% (21.1-33.8)<br>Diff 2014 vs 2008: 1.5% (-7.9-10.9%);<br><br>Adjusted OR (95% CI)<br>2008: 1<br>2010: 0.7 (0.42-1.16)<br>2012: 1.37 (0.87-2.17)<br>2014: 1.08 (0.67-1.75)<br><br>Linear Trend:<br>aOR = 1.05 (95% CI: 0.97-1.13)<br><br>*Adjusted for age, gender and ethnicity*<br><br>No mention of findings for current smokers. | **Neither hardening nor softening** evidenced by no significant change in the proportion of daily smokers who supported cigarettes and tobacco not being sold in 10 years' time | Good |

| AUSTRALIA: Dependence | | | | | | |
|---|---|---|---|---|---|---|
| **Publication details (author, publication year, survey years, number of years covered, number of data points, location, data source)** | **Smoking prevalence in total population** | **Measures** | **Key findings in total population** | **Key findings in smoking population** | **Hardening, softening or no change** | **Quality assessment** |
| **Clare et al (2014)[1]**<br><br>2001-2010; 10; 4<br><br>Australia<br><br>National Drug Strategy Household Survey | 2001: 22.4%<br>2004: 20.0 %<br>2007: 18.5%<br>2010: 19.0% | <u>Proportion of heavy smokers</u><br>Proportion of current smokers that were heavy smokers (≥ 15 cigarettes per day (CPD))<br><br>Current smokers: daily, weekly or less than weekly; aged ≥ 18 years | <u>Absolute Prevalence (%):</u><br>2001: 9.4%~<br>2010: 8.0%~ | <u>Proportion (%):</u><br>2001: 42.0%<br>2004: 42.4%<br>2007: 43.9%<br>2010: 41.2%<br><br>The proportion of smokers who reported heavy smoking was reported by the authors as consistent across the four waves. No statistical test reported.<br><br>*Adjusted for age, sex, SEIFA, education, and other sociodemographic variables* | **No statistical test reported** | Good |
| **Brennan et al (2019)[2]**<br><br>2001-2016; 16; 16<br><br>Victoria, Australia<br><br>The Victorian Smoking and Health survey | 2001: 20.1%<br>2016: 13.0%<br><br>aOR = 0.98 (95% CI: 0.98 to 0.99)<br><br>≥26 years of age | <u>Proportion of daily smokers</u><br>Proportion of current smokers that were daily smokers<br><br>Current smokers: smoked any tobacco, daily, weekly or less than weekly; aged ≥ 26 years | <u>Absolute prevalence (%):</u><br>2001: 16.9%~<br>2016: 10.4%~ | <u>Proportion (%)</u><br>2001: 84.2<br>2002: 87.3<br>2003: 88.1<br>2004: 85.2<br>2005: 87.4<br>2006: 85.3<br>2007: 84.1<br>2008: 84.5<br>2009: 83.7<br>2010: 83.4<br>2011: 81.9<br>2012: 82.9<br>2013: 86.0<br>2014: 81.7<br>2015: 76.8<br>2016: 79.7<br><br><u>Linear Trend:</u><br>aOR = 0.96 (95% CI: 0.95 to 0.98)<br>$p < 0.001$<br><br>*Adjusted for sex, age, education and SES* | **Softening** evidenced by statistically significant decrease in proportion of current smokers who were daily smokers | Good |

| **AUSTRALIA: Dependence** | | | | | | |
|---|---|---|---|---|---|---|
| **Publication details (author, publication year, survey years, number of years covered, number of data points, location, data source)** | **Smoking prevalence in total population** | **Measures** | **Key findings in total population** | **Key findings in smoking population** | **Hardening, softening or no change** | **Quality assessment** |
| **Brennan et al (2019)[2]**<br><br>2001-2016; 16; 16<br><br>Victoria, Australia<br><br>The Victorian Smoking and Health survey | | Proportion of heavy smokers<br>Proportion of current smokers that were heavy smokers (≥ 16 CPD)<br><br>Current smokers: smoked any tobacco, daily, weekly or less than weekly; aged ≧ 26 years | Absolute Prevalence (%):<br>2001: 8.5%~<br>2016: 2.8%~ | Proportion (%):<br>2001: 42.3<br>2002: 41.0<br>2003: 44.0<br>2004: 34.3<br>2005: 37.9<br>2006: 35.3<br>2007: 36.0<br>2008: 35.6<br>2009: 34.9<br>2010: 28.0<br>2011: 27.0<br>2012: 28.0<br>2013: 22.3<br>2014: 25.4<br>2015: 24.1<br>2016: 21.3<br><br>Linear Trend:<br>aOR = 0.93 (95% CI: 0.92 to 0.94)<br>$p < 0.001$<br><br>*Adjusted for sex, age, education and SES* | **Softening** evidenced by statistically significant decrease in proportion of current smokers who were heavy smokers | Good |

| INTERNATIONAL: Dependence | | | | | | |
|---|---|---|---|---|---|---|
| **Publication details (author, publication year, survey years, number of years covered, number of data points, location, data source)** | **Smoking prevalence in total population** | **Measures** | **Key findings in total population** | **Key findings in smoking population** | **Hardening, softening or no change** | **Quality assessment** |
| **Coady et al (2012)[6]**<br><br>2002-2008; 7; 2<br><br>New York City, US<br><br>NYC Community Health Survey | Prevalence (%) (95% CI)<br>2002: 21.5 (20.5-22.6)<br>2008: 15.8 (14.6-17.1)<br><br>Declined 27%, $p < 0.001$ | Mean cigarettes per day<br><br>Current smokers: at least 100 cigarettes in a lifetime and currently smoking on all or some days; ≥ 18 years of age | Not reported, unable to calculate | The mean CPD among daily smokers declined significantly from 14.6 (95% CI: 14.0-15.2) to 12.5 (95% CI: 11.6-13.4, $p < 0.001$) | **Softening** evidenced by statistically significant decrease in CPD | Fair |

| INTERNATIONAL: Dependence | | | | | | |
|---|---|---|---|---|---|---|
| **Publication details (author, publication year, survey years, number of years covered, number of data points, location, data source)** | **Smoking prevalence in total population** | **Measures** | **Key findings in total population** | **Key findings in smoking population** | **Hardening, softening or no change** | **Quality assessment** |
| **Coady et al (2012)[6]**<br><br>2002-2008; 7; 2<br><br>New York City, US<br><br>NYC Community Health Survey | | Heavy smoking decline compared to other smoking decline<br>The decline of heavy daily smoking prevalence compared to nondaily and light daily smoking prevalence<br><br>Current smokers: at least 100 cigarettes in a lifetime and currently smoking on all or some days; ≥ 18 years of age | Absolute prevalence (%, 95% CI)<br>*Daily smoking:*<br>2002:14.5 (13.6-15.4)<br>2008: 10.3 (9.3-11.3)<br>Declined 29%, p < 0.001.<br><br>*Heavy daily smoking (≥ 11 CPD):*<br>2002: 7.8 (7.1-8.5)<br>2008: 4.3 (3.7-4.9)<br>Declined 45%, p < 0.001<br><br>*Light daily smoking (1-10CPD):*<br>2002: 6.7 (6.1-7.4)<br>2008: 6.0 (5.2-6.8)<br>Declined 10.4%, p = 0.158<br><br>*Nondaily smoking:*<br>2002: 7.0 (6.4-7.7)<br>2008: 5.5 (4.8-6.4)<br>Declined 21%, p = 0.005 | Proportion (%):<br>*Daily smoking:*<br>2002: 67*<br>2008: 65*<br><br>*Heavy daily smoking:*<br>2002: 36*<br>2008: 27*<br><br>*Light daily smoking:*<br>2002: 31*<br>2008: 38*<br><br>*Nondaily smoking:*<br>2002: 33*<br>2008: 35* | **Softening,** as per the authors definition of hardening, evidenced by steeper decline in the prevalence of heavy daily smokers compared to light and nondaily smokers | Fair |

| INTERNATIONAL: Dependence | | | | | | |
|---|---|---|---|---|---|---|
| **Publication details (author, publication year, survey years, number of years covered, number of data points, location, data source)** | **Smoking prevalence in total population** | **Measures** | **Key findings in total population** | **Key findings in smoking population** | **Hardening, softening or no change** | **Quality assessment** |
| **Docherty et al (2014)[3]**<br><br>2000-2010; 11; 11<br><br>England<br><br>UK General Lifestyle Survey | 2000: 27%<br>2010: 20%<br>(≥ 16 years of age) | <u>Time to first cigarette (TTFC)</u><br>Proportion of current smokers, ≥ 26 years of age, with TTFC ≤ 30 minutes after waking<br><br>*Q. 'How soon after waking do you usually smoke your first cigarette?' A.'30 minutes or less'* | Not reported, unable to calculate | <u>Proportion (estimated):</u><br>2000: ~46%[+]<br>2010: ~46%[+]<br><br><u>aOR (95% CI):</u><br>2000: 1.00<br>2001: 1.04 (0.93–1.16)<br>2002: 1.06 (0.95–1.18)<br>2003: 1.08 (0.97–1.20)<br>2004: 1.12 (1.00–1.25)<br>2005: 1.01 (0.91–1.12)<br>2006: 1.05 (0.94–1.17)<br>2007: 1.08 (0.96–1.21)<br>2008: 1.10 (0.97–1.23)<br>2009: 1.12 (0.99–1.26)<br>2010: 1.02 (0.90–1.16)<br><br>No significant trend over time for TTFC ≤30 mins, p(trend)=0.288<br><br>*Adjusted for age, sex, socioeconomic status* | **Neither hardening nor softening** evidenced by no statistically significant change in the proportion of smokers having their first cigarette 30 minutes or less after waking | Good |

| INTERNATIONAL: Dependence | | | | | | |
|---|---|---|---|---|---|---|
| **Publication details (author, publication year, survey years, number of years covered, number of data points, location, data source)** | **Smoking prevalence in total population** | **Measures** | **Key findings in total population** | **Key findings in smoking population** | **Hardening, softening or no change** | **Quality assessment** |
| **Docherty et al (2014)[3]**<br><br>2000-2010; 11; 11<br><br>England<br><br>Health Survey for England | 2000: 26%[#]<br>2010: 20%[#]<br>(≥ 16 years of age) | Time to first cigarette:<br>Proportion of current smokers, ≥ 26 years of age, with TTFC ≤ 30 minutes after waking<br><br>*Q. 'How soon after waking do you usually smoke your first cigarette?'*<br>*A. '30 minutes or less'* | Not reported, unable to calculate | Proportion (estimated)<br>2000: ~46%[+]<br>2010: ~48%[+]<br><br>aOR (95% CI):<br>2000: 1.00<br>2001: 1.07 (0.95-1.20)<br>2002: 1.15 (1.00-1.32)<br>2003: 1.23 (1.09-1.38)<br>2004: 1.18 (1.02-1.36)<br>2005: 1.21 (1.05-1.39)<br>2006: 1.16 (1.02-1.31)<br>2007: 1.00 (0.86-1.16)<br>2008: 1.13 (1.00-1.28)<br>2009: 1.29 (1.09-1.52)<br>2010: 0.70 (0.56-0.86)<br><br>No significant trend over time for TTFC ≤ 30mins, p(trend)=0.785<br><br>*Adjusted for age, sex, socioeconomic status* | **Neither hardening nor softening** evidenced by no statistically significant change in the proportion of smokers having their first cigarette 30 minutes or less after waking | Good |

| INTERNATIONAL: Dependence | | | | | | |
|---|---|---|---|---|---|---|
| **Publication details (author, publication year, survey years, number of years covered, number of data points, location, data source)** | **Smoking prevalence in total population** | **Measures** | **Key findings in total population** | **Key findings in smoking population** | **Hardening, softening or no change** | **Quality assessment** |
| **Smith et al (2014)**[7]<br><br>2002-2012; 11; 11<br><br>US<br><br>National Survey on Drug Use and Health (NSDUH; formerly the National Household Survey on Drug Abuse) | Consistently declined throughout 2002–2012, from 23.6% to 18.9%. | Proportion of heavy smokers<br>Proportion of all current smokers that were heavy smokers (≥ 25 CPD)<br><br>Current smoker: ≥ 100 cigarettes in their lifetime and at least once during the past 30 days; ≥ 12 years of age | Absolute Prevalence (%):<br>2002: 2.1%*<br>2012: 1.1%* | Among those who smoked, the proportion of heavy smokers declined, from 8.8% to 5.6% | **No statistical test reported** | Fair |
| **Smith et al (2014)**[7]<br><br>2002-2012; 11; 11<br><br>US<br><br>National Survey on Drug Use and Health (NSDUH; formerly the National Household Survey on Drug Abuse) | | Nicotine dependence severity<br>Nicotine Dependence Syndrome Scale<br><br>Current smokers: 100+ cigarettes in their lifetime and at least once during the past 30 days; ≥ 12 years of age | Not applicable | There is a small decline in the overall nicotine dependence severity over time (the slope for a linear time variable was −0.005 (95% CI:−0.007, −0.004; $p < 0.001$). There was no significant change in variability for the nicotine dependence severity measure over the study period | **Softening** evidenced by a statistically significant decrease in Nicotine Dependence Syndrome Scale scores | Fair |

| INTERNATIONAL: Dependence | | | | | | |
|---|---|---|---|---|---|---|
| **Publication details (author, publication year, survey years, number of years covered, number of data points, location, data source)** | **Smoking prevalence in total population** | **Measures** | **Key findings in total population** | **Key findings in smoking population** | **Hardening, softening or no change** | **Quality assessment** |
| **Azagaba (2015)[8]**<br><br>2004-2012; 9; 9<br><br>Canada<br><br>Canadian Tobacco Use and Monitoring Survey (CTUMS) | 2004: 19.6%<br>2012: 16.1%<br><br>(current smokers, ≥ 15 years) | <u>Time-to-first cigarette (TTFC)</u><br>Proportion of current daily smokers that reported TTFC ≤ 5 minutes and/or TTFC ≤ 30 minutes after waking<br><br>Current daily smokers: smoked every day and had smoked 100 cigarettes in their lifetime; ≥ 26 years of age | Not reported, unable to be calculated | <u>Survey Year</u> <u>TTFC ≤ 30 min aOR(95% CI)</u> <u>TTFC ≤ 5 min aOR(95% CI)</u><br>2004: ref ref<br>2005: 1.24 (0.95–1.63) 1.08 (0.80–1.45)<br>2006: 0.88 (0.69–1.14) 0.89 (0.66–1.19)<br>2007: 1.19 (0.92–1.53) 1.08 (0.82–1.43)<br>2008: 1.18 (0.91–1.54) 1.11 (0.83–1.48)<br>2009: 0.90 (0.69–1.17) 0.94 (0.71–1.25)<br>2010: 0.94 (0.72–1.24) 0.72 (0.54–0.97)<br>2011: 0.89 (0.68–1.17) 0.93 (0.70–1.26)<br>2012: 1.28 (0.95–1.71) 1.02 (0.72–1.44)<br><br>There was no statistically significant difference between survey years when compared with the reference or base year, 2004, except for TTFC ≤ 5 min after waking 2010 vs 2004 (ref) aOR (95% CI): 0.72 (0.54-0.97)<br><br>*Adjusted for age, sex, educational level, employment status, marital status, and province of residence.* | **Neither hardening nor softening** evidenced by no statistically significant change in time to first cigarette after waking | Good |
| **Kulik and Glantz (2016)[4]**<br><br>1992/1993-2011/2012; 18; 302 (7 waves)<br><br>Note: not all states had data for all years.<br><br>US<br><br>Tobacco Use Supplement of the Current Population Survey (TUS-CPS) | Not reported | <u>Cigarettes per day</u><br><br>Current smokers (smoked at least 100 cigarettes in lifetime and currently smoking every day or for some days), ≥ 18 years of age | Not reported | Consumption drops by 0.32 CPD (±0.02 SE, $p < 0.001$) for each 1.00% drop in smoking prevalence | **Softening** evidenced by a statistically significant positive association between prevalence and smoking prevalence | Fair |

| INTERNATIONAL: Dependence | | | | | | |
|---|---|---|---|---|---|---|
| **Publication details (author, publication year, survey years, number of years covered, number of data points, location, data source)** | **Smoking prevalence in total population** | **Measures** | **Key findings in total population** | **Key findings in smoking population** | **Hardening, softening or no change** | **Quality assessment** |
| **Edwards et al (2017)[5]**<br><br>2008-2014; 7; 4<br><br>New Zealand<br><br>The Health and Lifestyle Survey (HLS) | Daily Smoking Prevalence (%) (95% CI)<br>2008: 16.9 (14.3-19.5)<br><br>2010: 15.8 (12.8-18.8)<br><br>2012: 14.6 (12.9-16.4)<br><br>2014: 14.4 (12.4-16.4)<br><br>Difference 2014 vs 2008 (95% CI): -2.5% (-5.8- 0.8) | Proportion daily smokers<br>Proportion daily smokers among current smokers (smoke at least once a month), ≥ 15 years of age | Absolute Prevalence (%):<br>2008: 15.2%~<br>2017: 11.4% ~ | Proportion (%) (95% CI)<br>2008: 89.9 (85.6-94.2)<br>2010: 88.9 (83.8-94.1)<br>2012: 92.1 (89.1-95.2)<br>2014: 92.5 (89.6-95.5)<br>Diff 2014 vs 2008: 2.6% (95% CI: -2.4-7.7)<br><br>Adjusted OR (95% CI):<br>2008: 1.0<br>2010: 0.90 (0.440-1.82)<br>2012: 1.32 (0.70-2.51)<br>2014: 1.41 (0.75-2.64)<br><br>Linear Trend:<br>aOR = 1.07 (95% CI: 0.97-1.18)<br><br>*AOR adjusted for age, gender and ethnicity. Proportion adjusted for age.* | **Neither hardening nor softening** evidenced by no statistically significant change in the proportion of current smokers who were daily smokers | Good |

| INTERNATIONAL: Dependence | | | | | | |
|---|---|---|---|---|---|---|
| **Publication details (author, publication year, survey years, number of years covered, number of data points, location, data source)** | **Smoking prevalence in total population** | **Measures** | **Key findings in total population** | **Key findings in smoking population** | **Hardening, softening or no change** | **Quality assessment** |
| **Edwards et al (2017)[5]**<br><br>2008-2014; 7; 4<br><br>New Zealand<br><br>The Health and Lifestyle Survey (HLS) | | Proportion daily smokers with 4 or more quit attempts<br>The proportion of daily smokers who had made four or more quit attempts of more than 24 hours in the past year, ≥ 15 years of age | Absolute Prevalence (%):<br>2008: 0.83%~<br>2014: 0.96%~ | Proportion (%) (95% CI):<br>2008: 4.9 (1.8-8.0)<br>2010: 7.5 (3.5-11.4)<br>2012: 9.9 (5.3-14.5)<br>2014: 6.7 (3.6-9.8)<br><br>Diff 2014 vs 2008: 1.8% (95% CI: -2.4-6.1)<br><br>Adjusted OR (95% CI):<br>2008: 1.0<br>2010: 1.56 (0.67, 3.63)<br>2012: 2.11 (0.94, 4.74)<br>2014: 1.40 (0.62, 3.17)<br><br>Linear Trend:<br>aOR = 1.05 (95% CI: 0.95-1.17)<br><br>*AOR adjusted for age, gender and ethnicity. Proportion adjusted for age.*<br><br>Findings similar to current smokers | **Neither hardening nor softening** evidenced by no statistically significant change in the proportion of current smokers who had made four or more quit attempts of more than 24 hours in the past year | Good |

| INTERNATIONAL: Dependence | | | | | | |
|---|---|---|---|---|---|---|
| **Publication details (author, publication year, survey years, number of years covered, number of data points, location, data source)** | **Smoking prevalence in total population** | **Measures** | **Key findings in total population** | **Key findings in smoking population** | **Hardening, softening or no change** | **Quality assessment** |
| **Goodwin et al (2018)[9]**<br><br>2002-2015; 14; 14<br><br>US<br><br>National Survey on Drug Use and Health | In 2002, the smoking prevalence was 26%. This declined to 19.48% in 2015.<br><br>Linear Trend - unadjusted OR (95% CI): 0.72 (0.70 to 0.74) | Proportion of heavy smokers<br>Proportion current smokers (smoked part or all of a cigarette during the past 30 days), > 12 years of age, that are heavy smokers (16+ CPD) | Absolute Prevalence (%):<br>2002: 9.82%~<br>2015: 5.37%~ | Proportion (%):<br>Year 1-5 CPD 6-15 CPD ≥16 CPD<br>2002: 37.00 25.25 37.75<br>2003: 37.88 24.99 37.12<br>2004: 37.39 25.16 37.45<br>2005: 37.17 27.16 35.66<br>2006: 37.57 27.38 35.05<br>2007: 38.68 27.09 34.23<br>2008: 39.38 26.79 33.83<br>2009: 40.81 27.48 31.71<br>2010: 41.98 28.41 29.61<br>2011: 41.58 28.78 29.64<br>2012: 42.36 28.24 29.41<br>2013: 43.17 29.09 27.74<br>2014: 43.29 29.66 27.05<br>2015: 43.64 28.77 27.59<br><br>Odds Ratio for Linear Trend (95% CI):<br>1-5 CPD: 1.37 (1.30-1.45, p < 0.0001)<br>6-15 CPD: 1.25 (1.18-1.32, p < 0001)<br>≥ 16 CPD: 0.58 (0.55-0.61, p < 0.0001)<br><br>The proportion of those smoking 1-5 CPD and 6-15 CPD increased significantly between 2002 and 2015. The proportion of current smokers smoking ≥ 16 CPD decreased significantly. | **Softening** evidenced by statistically significant decrease in heavy smokers (≥ 16 CPD) | Good |

| **INTERNATIONAL: Dependence** | | | | | | |
|---|---|---|---|---|---|---|
| **Publication details (author, publication year, survey years, number of years covered, number of data points, location, data source)** | **Smoking prevalence in total population** | **Measures** | **Key findings in total population** | **Key findings in smoking population** | **Hardening, softening or no change** | **Quality assessment** |
| **Goodwin et al (2018)**[9]<br><br>2002-2015; 14; 14<br><br>US<br><br>National Survey on Drug Use and Health | | <u>Time to first cigarette</u> <u>(TTFC):</u><br>Proportion of current smokers who report time to first cigarette <30 min after waking in the morning<br><br>Current smokers: smoked part or all of a cigarette during the past 30 days; ≥ 12 years of age | <u>Absolute Prevalence (%):</u><br>2002: 13.11%*<br>2015: 9.18 %* | <u>Proportion (%):</u><br>2002: 50.42<br>2003: 50.01<br>2004: 50.58<br>2005: 48.15<br>2006: 48.32<br>2007: 47.94<br>2008: 48.58<br>2009: 47.33<br>2010: 46.75<br>2011: 48.72<br>2012: 47.35<br>2013: 47.39<br>2014: 47.77<br>2015: 47.13<br><br><u>Linear Trend:</u><br>OR=0.89 (95% CI: 0.84-0.94), p(trend) < 0.0001<br>aOR = 1.18 (95%CI: 1.10-1.26), p(trend) < 0.0001<br><br>*Adjusted for CPD, age, gender and family income* | **Softening** evidenced by a statistically significant decrease in the prevalence of smokers that have their first cigarette less than 30 minutes after waking before adjustment.<br><br>Following adjustment for CPD and other covariates, authors report observing **hardening**. | Good |

| AUSTRALIA: Hardcore smoking (combination of dependence and motivational measures) | | | | | | |
|---|---|---|---|---|---|---|
| **Publication details; Survey years, number of years covered, number of data points; Data source; location** | **Smoking Prevalence in total population** | **Measure** | **Key Findings in Total Population** | **Key findings in Smoking Population** | **Hardening, softening or no change** | **Quality Assessment** |
| **Clare et al (2014)**[1]<br><br>2001 to 2010; 10; 4<br><br>Australia<br><br>National Drug Strategy Household Survey | 2001: 22.4%<br>2004: 20.0 %<br>2007: 18.5%<br>2010: 19.0% | Proportion of current smokers that were hardcore smokers<br><br>Hardcore Smokers: smoked more than 15 CPD (heavy smokers), had made no quit attempt in the last 12 months, and did not intend to quit.<br><br>Current smokers: smoked daily, weekly or less than weekly; aged ≥ 18 years | Absolute Prevalence (%):<br>2001: 2.5%<br>2004: 2.2%<br>2007: 2.2%<br>2010: 2.0% | Proportion (%):<br>2001: 11.9%<br>2004: 10.9%<br>2007: 11.8%<br>2010: 10.7%<br><br>Adjusted OR (95% CI):<br>2001: 1.00 (ref)<br>2004: 0.95 (0.78-1.15)<br>2007: 1.10 (0.89-1.36)<br>2010: 0.95 (0.78-1.17)<br>p = 0.550<br><br>*Adjusted for age, sex, SEIFA, education, and other sociodemographic variables.* | **Neither hardening nor softening** evidenced by no significant change in the proportion of smokers that were hardcore | Good |

| AUSTRALIA: Hardcore smoking (combination of dependence and motivational measures) | | | | | | |
|---|---|---|---|---|---|---|
| **Publication details; Survey years, number of years covered, number of data points; Data source; location** | **Smoking Prevalence in total population** | **Measure** | **Key Findings in Total Population** | **Key findings in Smoking Population** | **Hardening, softening or no change** | **Quality Assessment** |
| **Brennan et al (2019)[2]**<br><br>2001-2016; 16; 16<br><br>Victoria, Australia<br><br>The Victorian Smoking and Health survey | 2001: 20.1%<br>2016: 13.0%<br><br>aOR = 0.98 (95% CI: 0.98 to 0.99)<br><br>(≥ 26 years of age) | Proportion of current smokers that were hardcore smokers<br><br>Hardcore smokers: smoked every day, smoked ≥ 16 CPD (heavy consumption), had not made a quit attempt within the past 12 months, and did not intend to quit within the next 6 months.<br><br>Current smokers: smoked any tobacco, daily, weekly or less than weekly; aged ≥ 26 years<br><br><u>*Quit Attempts*</u><br>*Q. 'Approximately how many times, if any, have you tried to give up smoking?'*<br>*Q. 'How long ago did you last attempt to quit smoking end?'*<br><br><u>*Quit Intention*</u><br>*Q. 'Are you seriously considering quitting smoking in the next 6 months?'*<br><br>Sensitivity analyses with different definitions were also undertaken | <u>Absolute Prevalence (%):</u><br>2001: 3.5%~<br>2016: 1.2%<br>(≥ 26 years of age) | <u>Proportion (%):</u><br>2001: 17.2%<br>2002: 15.3%<br>2003: 16.6%<br>2004: 13.0%<br>2005: 17.0%<br>2006: 13.3%<br>2007: 13.3%<br>2008: 10.9%<br>2009: 14.4%<br>2010: 11.3%<br>2011: 9.0%<br>2012: 9.8%<br>2013: 5.8%<br>2014: 10.1%<br>2015: 8.3%<br>2016: 9.1%<br><br><u>Linear Trend:</u><br>aOR = 0.94 (95% CI: 0.92-0.96)<br>$p < 0.001$<br><br>*Adjusted for sex, age, education and SES*<br><br><u>Sensitivity analysis:</u><br>Irrespective of the definition of hardcore smokers, the prevalence declined significantly. | **Softening** evidenced by a significant decrease in the proportion of smokers that were hardcore | Good |

| AUSTRALIA: Hardcore smoking (combination of dependence and motivational measures) | | | | | | |
|---|---|---|---|---|---|---|
| **Publication details; Survey years, number of years covered, number of data points; Data source; location** | **Smoking Prevalence in total population** | **Measure** | **Key Findings in Total Population** | **Key findings in Smoking Population** | **Hardening, softening or no change** | **Quality Assessment** |
| **Brennan et al (2019)[2]**<br><br>2001-2016; 16; 16<br><br>Victoria, Australia<br><br>The Victorian Smoking and Health survey | | Proportion of current smokers who had 'given up giving up' among current smokers<br><br>Given up giving up: smoked every day, had previously made ≥ 5 quit attempts, had not made a quit attempt within the past 5 years, and did not intend to quit within the next 6 months.<br><br>Current smokers: smoked any tobacco, daily, weekly or less than weekly), aged ≥ 26 years<br><br><u>Quit Attempts</u><br>Q. 'Approximately how many times, if any, have you tried to give up smoking?'<br>Q. 'How long ago did you last attempt to quit smoking end?'<br><br><u>Quit Intention</u><br>Q. 'Are you seriously considering quitting smoking in the next 6 months?' | <u>Absolute Prevalence (%):</u><br>2001: 0.2%~<br>2016: 0.1%~ | <u>Proportion (%):</u><br>2001: 1.1%<br>2002: 0.5%<br>2003: 1.4%<br>2004: 0.6%<br>2005: 1.1%<br>2006: 0.4%<br>2007: 0.6%<br>2008: 0.9%<br>2009: 0.5%<br>2010: 0.6%<br>2011: 0.8%<br>2012: 1.1%<br>2013: 0.4%<br>2014: 0.7%<br>2015: 0.5%<br>2016: 0.6%<br><br>Significance testing not conducted due to low probability outcome | **No statistical test conducted** | **Good** |

| INTERNATIONAL: Hardcore smoking (combination of dependence and motivational measures) | | | | | | |
|---|---|---|---|---|---|---|
| **Publication details; Survey years, number of years covered, number of data points; Data source; location** | **Smoking Prevalence in total population** | **Measure** | **Key Findings in Total Population** | **Key findings in Smoking Population** | **Hardening, softening or no change** | **Quality Assessment** |
| **Lund et al (2011)[10]**<br><br>1996-2009; 14; 14<br><br>Norway<br><br>Statistics Norway population database | 1996: 46%*<br>2009: 27%*<br><br>(25 to 74 years of age)<br><br>*The authors note that in the adult population there has been no significant decline in smoking prevalence.* | Proportion of smokers who were daily hardcore smokers<br><br>Daily Hardcore smoker:<br>Smokers with no quit attempt during the previous 12 months, no intention to quit in the next 6 months, and a belief in continued smoking status in five years.<br><br>Smokers: daily and occasional smokers; aged 25-74 years<br><br>*<u>Quit Intention:</u>*<br>*Q. 'Are you considering to quit during the next six months?'*<br><br>*<u>Future Smoking Status:</u>*<br>*Q. 'Try to predict your smoking status in five years from now. Which statement fits your belief best?'*<br>*A. (a)* ***'I will definitely be a daily smoker'****, (b)* ***'I will probably be a daily smoker'****, (c) 'I will probably not be a daily smoker', (d) 'I will definitely not be a daily smoker', or* ***'I don't know'****.*<br><br>*<u>Quit Attempt</u>*<br>*Q. Have you tried to quit smoking during the latest 12 months?* | <u>Absolute Prevalence (%):</u><br>1996: 14%<br>1997: 16%<br>1998: 16%<br>1999: 15%<br>2000: 12%<br>2001: 13%<br>2002: 12%<br>2003: 9%<br>2004: 8%<br>2005: 7%<br>2006: 9%<br>2005: 7%<br>2008: 7%<br>2009: 6%<br><br>(aged 25-74 years) | <u>Proportion (%):</u><br>1996/1997: 32.6%*<br>1998/1999: 36.7%*<br>2000/2001: 31.0%*<br>2002/2003: 28.6%*<br>2004/2005: 21.1%*<br>2006/2007: 26.5%*<br>2008/2009: 23.6%*<br><br><u>Adjusted OR (95% CI):</u><br>1996/1997: 1.00 (ref)<br>1998/1999: 1.25 (1.02-1.52)<br>2000/2001: 0.92 (0.75-1.13)<br>2002/2003: 0.81 (0.66-1.00)<br>2004/2005: 0.55 (0.43-0.70)<br>2006/2007: 0.71 (0.57-0.89)<br>2008/2009: 0.59 (0.40-0.72)<br><br>Survey year (1-7), OR=0.90 (95% CI: 0.88-0.93)<br><br>*Adjusted for gender, age, educational level, and the use of snus (smokeless tobacco)* | **Softening** evidenced by a significant decrease in the proportion of smokers that were hardcore | Good |
| **Docherty et al (2014)[3]**<br><br>2000-2010; 11; 11<br><br>England | 2000: 27%<br>2010: 20%<br>(≥ 16 years of age) | Prevalence of current smokers ≥ 26 years of age who were hardcore smokers<br><br>Hardcore smoker: low motivation (do not want to quit) and highly | Not reported, unable to calculate | <u>Proportion (estimated):</u><br>2000: ~12%⁺<br>2010: ~13%⁺<br><br><u>Adjusted OR (95% CI):</u><br>2000: 1.00 | **Hardening** evidenced by a statistically significant increase in the proportion of | Good |

| INTERNATIONAL: Hardcore smoking (combination of dependence and motivational measures) | | | | | | |
|---|---|---|---|---|---|---|
| **Publication details; Survey years, number of years covered, number of data points; Data source; location** | **Smoking Prevalence in total population** | **Measure** | **Key Findings in Total Population** | **Key findings in Smoking Population** | **Hardening, softening or no change** | **Quality Assessment** |
| General Lifestyle Survey | | dependent (have their first cigarette within 30 minutes of waking)<br><br>*Low motivation to quit:*<br>*Q. 'would you like to give up smoking altogether?'*<br>*A. 'No'*<br><br>*Highly dependent:*<br>*Q. 'How soon after waking do you usually smoke*<br>*your first cigarette?*<br>*A. '30 minutes or less'* | | 2001: 1.04 (0.88–1.23)<br>2002: 1.09 (0.92–1.29)<br>2003: 1.21 (1.03–1.42)<br>2004: 1.10 (0.93–1.30)<br>2005: 1.09 (0.93–1.28)<br>2006: 1.03 (0.86–1.22)<br>2007: 1.26 (1.06–1.50)<br>2008: 1.36 (1.15–1.62)<br>2009: 1.30 (1.09–1.55)<br>2010: 1.21 (1.01–1.45)<br><br>p(trend) < 0.001<br><br>*Adjusted for age, sex, socioeconomic status* | smokers that were hardcore | |
| **Docherty et al (2014)**[3]<br><br>2000-2010; 11; 11<br><br>England<br><br>Health Survey for England | 2000: 26%[#]<br>2010: 20%[#]<br>(≥ 16 years of age) | Prevalence of current smokers, ≥ 26 years of age, who were hardcore smokers<br><br>Hardcore smoker definition: low motivation (do not want to quit) and highly dependent (have their first cigarette within 30 minutes of waking)<br><br>*Low motivation to quit:*<br>*Q. 'would you like to give up smoking altogether?'*<br>*A. 'No'*<br><br>*Highly dependent:*<br>*Q. 'How soon after waking do you usually smoke*<br>*your first cigarette?*<br>*A. '30 minutes or less'* | Not reported, unable to calculate | <u>Proportion (estimated):</u><br>2000: ~13%[+]<br>2010: ~15%[+]<br><br><u>Adjusted OR (95% CI):</u><br>2000: 1.00 (ref)<br>2001: 1.08 (0.91-1.29)<br>2002: 1.23 (1.01-1.49)<br>2003: 1.12 (0.94-1.33)<br>2004: 1.16 (0.94-1.43)<br>2005: 1.11 (0.91-1.36)<br>2006: 1.18 (0.99-1.40)<br>2007: 1.11 (0.90-1.37)<br>2008: 1.33 (1.12-1.58)<br>2009: 1.35 (1.08-1.70)<br>2010: 0.73 (0.55-0.96)<br><br>p(trend) = 0.040<br><br>*Adjusted for age, sex, socioeconomic status* | **Hardening** evidenced by a statistically significant increase in the proportion of smokers that were hardcore | Good |

| INTERNATIONAL: Hardcore smoking (combination of dependence and motivational measures) | | | | | | |
|---|---|---|---|---|---|---|
| **Publication details; Survey years, number of years covered, number of data points; Data source; location** | **Smoking Prevalence in total population** | **Measure** | **Key Findings in Total Population** | **Key findings in Smoking Population** | **Hardening, softening or no change** | **Quality Assessment** |
| **Azagba (2015)**[8]<br><br>2004 to 2012; 9; 9<br><br>Canada<br><br>Canadian Tobacco Use and Monitoring Survey (CTUMS) | 2004: 19.6%*<br>2012: 16.1%*<br>(current smokers, ≥ 15 years of age) | Prevalence of current daily smokers who were hardcore smokers<br><br>Hardcore Smoker<br>Definition 1: no quit attempt lasting for at least 24 hours in the last 12 months AND no intention to quit in the next 6 months AND time to first cigarette (TTFC) < 30 minutes.<br><br>Current daily smokers: smoked every day and had smoked 100 cigarettes in their lifetime; ≥ 26 years of age<br><br>*Questions not reported* | Not reported, unable to calculate | **Definition 1:**<br>Proportion:<br>19.7% of daily smokers<br><br>aOR (95%CI)<br>2004: Ref<br>2005:0.95 (0.70–1.30)<br>2006: 0.77 (0.56–1.06)<br>2007: 0.92 (0.68–1.24)<br>2008: 1.15 (0.85–1.57)<br>2009: 0.96 (0.70–1.31)<br>2010: 0.83 (0.60–1.15)<br>2011: 0.71 (0.52–0.97)<br>2012: 1.10 (0.79–1.52)<br>Test for trend not statistically significant (but not reported)<br><br>*Adjusted for age, sex, educational level, employment status, marital status, and province of residence* | **Neither hardening nor softening** evidenced by no significant change in the proportion of smokers that were hardcore | Good |

| INTERNATIONAL: Hardcore smoking (combination of dependence and motivational measures) | | | | | | |
|---|---|---|---|---|---|---|
| **Publication details; Survey years, number of years covered, number of data points; Data source; location** | **Smoking Prevalence in total population** | **Measure** | **Key Findings in Total Population** | **Key findings in Smoking Population** | **Hardening, softening or no change** | **Quality Assessment** |
| **Azagba (2015)**[8]<br><br>2004 to 2012; 9; 9<br><br>Canada<br><br>Canadian Tobacco Use and Monitoring Survey (CTUMS) | 2004: 19.6%*<br>2012: 16.1%*<br>(current smokers, ≥ 15 years of age) | Prevalence of current daily smokers who were hardcore smokers<br><br>Hardcore Smoker<br>Definition 1: no quit attempt lasting for at least 24 hours in the last 12 months AND no intention to quit in the next 6 months AND time to first cigarette (TTFC) < 30 minutes.<br><br>Definition 2: Definition 1 + smoked ≥ 15 CPD<br><br>Current daily smokers: smoked every day and had smoked 100 cigarettes in their lifetime; ≥ 26 years of age<br><br>*Questions not reported* | Not reported, unable to calculate | **Definition 2:**<br>Proportion:<br>14.3% of daily smokers<br><br>aOR (95% CI):<br>2004: Ref<br>2005: 1.00 (0.70–1.41)<br>2006: 0.90 (0.63–1.30)<br>2007: 1.01 (0.72–1.42)<br>2008: 1.11 (0.78–1.59)<br>2009: 0.97 (0.68–1.38)<br>2010: 0.91 (0.63–1.30)<br>2011: 0.72 (0.50–1.03)<br>2012: 1.04 (0.72–1.51)<br>Test for trend not statistically significant (but not reported)<br><br>*Adjusted for age, sex, educational level, employment status, marital status, and province of residence* | **Neither hardening nor softening** evidenced by no significant change in the proportion of smokers that were hardcore | Good |

| INTERNATIONAL: Quit Outcomes – Quit ratios/rates | | | | | | |
|---|---|---|---|---|---|---|
| **Publication details; Survey years, number of years covered, number of data points; Data source; location** | **Smoking prevalence in total population** | **Measure** | **Smoker definition** | **Key findings** | **Hardening, softening or no change** | **Quality assessment** |
| **Kulik and Glantz (2016)[4]**<br><br>1992/1993-2010/2011; 18; 357 (7 waves)<br><br>US<br>Tobacco Use Supplement of the Current Population Survey (TUS-CPS) | Not reported | Quit ratio<br>The percentage of ever smokers who have quit (quit ratio) was calculated by dividing the number of former smokers by the number of current and former smokers | Former smokers: persons who reported ever smoking ≥ 100 cigarettes but who do not currently smoke.<br><br>Current smokers: Persons ≥ 18 years of age who reported ever smoking ≥ 100 cigarettes and who currently smoke every day or on some days AND persons who reported ever smoking ≥ 100 cigarettes but who do not currently smoke | Quit ratio increased by 1.13% (±0.06 SE, $p < 0.001$) for each 1% decrease in smoking prevalence | **Softening** evidenced by a significant negative association between quit ratio and smoking prevalence | Fair |
| **Edwards et al (2017)[5]**<br><br>2008-2014; 7; 4<br><br>New Zealand<br><br>The Health and Lifestyle Survey (HLS). | Daily Smoking Prevalence (%) (95% CI):<br>2008: 16.9 (14.3-19.5)<br><br>2014: 14.4 (12.4-16.4)<br><br>Difference 2014 vs 2008 (95% CI):<br>-2.5% (-5.8-0.8) | Recent quit rate<br>Defined as the proportion of recent quitters within the past 1-12 months among the population of smokers eligible to quit in past year (=number of ex-smokers who quit 1-12 months ago/(number of ex-smokers who quit 0-12 months ago + number daily smokers)); quitters within the past month were excluded from the denominator due to the very short time since quitting (very high risk of relapse)<br><br>Current smokers (smoke at least once a month) and daily smokers, ≥ 15 years of age | NA | Proportion (%) (95% CI)<br>2008: 8.4 (4.1-12.7)<br>2010: 9.4 (4.6-14.1)<br>2012: 11.3 (7.3-15.3)<br>2014: 9.5 (4.5-14.4)<br>diff 2014 vs 2008: 1.1% (95% CI: -5.2-7.3)<br><br>aOR (95% CI)<br>2008: 1.0<br>2010: 1.11 (0.50 to 2.46)<br>2012: 1.38 (0.70 to 2.73)<br>2014: 1.14 (0.53 to 2.46)<br><br>Linear Trend:<br>aOR = 1.03 (95% CI: 0.92-1.15)<br><br>*Adjusted for age, gender and ethnicity*<br><br>Similar pattern for current smokers (data not shown) | **Neither hardening nor softening** evidenced by no statistically significant change in recent quit rate | Good |

| INTERNATIONAL: Quit Outcomes – Quit ratios/rates | | | | | | |
|---|---|---|---|---|---|---|
| **Publication details; Survey years, number of years covered, number of data points; Data source; location** | **Smoking prevalence in total population** | **Measure** | **Smoker definition** | **Key findings** | **Hardening, softening or no change** | **Quality assessment** |
| **Edwards et al (2017)[5]**<br><br>2008-2014; 7; 4<br><br>New Zealand<br><br>The Health and Lifestyle Survey (HLS). | Daily Smoking Prevalence (%) (95% CI)<br>2008: 16.9 (14.3-19.5)<br>2014: 14.4 (12.4-16.4)<br><br>Difference 2014 vs 2008: -2.5% (95% CI: -5.8-0.8) | Recent sustained quit rate<br>Defined as smokers who quit within the past 13-24 months as a proportion of the population eligible to quit in past 2 years (=number ex-smokers who quit 13-24 months ago/(number of ex-smokers who quit 0-24 months ago + number daily smokers))<br><br>Current smokers (smoke at least once a month) and daily smokers, ≥ 15 years of age | NA | Proportion (%) (95% CI)<br>2008: 6.9 (2.6-11.3)<br>2010: 7.4 (2.9-11.9)<br>2012: 6.9 (4.0-9.8)<br>2014: 12.4 (7.2-17.7)<br>diff 2014 vs 2008: 5.5% (-1.8-12.8)<br><br>aOR (95% CI)<br>2008: 1.00<br>2010: 1.05 (0.41-2.73)<br>2012: 0.98 (0.43-2.26)<br>2014: 1.88 (0.78-4.54)<br><br>Linear Trend:<br>aOR = 1.12 (95% CI: 0.96-1.30)<br><br>*Adjusted for age, gender and ethnicity*<br><br>Similar pattern for current smokers (data not shown). | **Neither hardening nor softening** evidenced by no statistically significant change in recent sustained quit rate | Good |

*Figure not reported by authors. Figures calculated by the authors of the current report.

~Figure not reported by authors. Figures calculated by the authors of the current report. Prevalence of the hardening indicator was weighted and/or adjusted but it is unclear if smoking prevalence was also weighted and/or adjusted.

+ Estimated from a graph presented in the study.

#Figure not reported by authors. Obtained by reviewing the original survey data source.36

## References


1. Clare P, Bradford D, Courtney RJ, Martire K, Mattick RP. The relationship between socioeconomic status and 'hardcore' smoking over time--greater accumulation of hardened smokers in low-SES than high-SES smokers. *Tob Control* 2014; **23**(e2): e133-8.
2. Brennan E, Greenhalgh EM, Durkin SJ, Scollo MM, Hayes L, Wakefield MA. Hardening or softening? An observational study of changes to the prevalence of hardening indicators in Victoria, Australia, 2001-2016. *Tob Control* 2019.
3. Docherty G, McNeill A, Gartner C, Szatkowski L. Did hardening occur among smokers in England from 2000 to 2010? *Addiction* 2014; **109**(1): 147-54.
4. Kulik MC, Glantz SA. The smoking population in the USA and EU is softening not hardening. *Tob Control* 2016; **25**(4): 470-5.
5. Edwards R, Tu D, Newcombe R, Holland K, Walton D. Achieving the tobacco endgame: evidence on the hardening hypothesis from repeated cross-sectional studies in New Zealand 2008-2014. *Tob Control* 2017; **26**(4): 399-405.
6. Coady MH, Jasek J, Davis K, Kerker B, Kilgore EA, Perl SB. Changes in smoking prevalence and number of cigarettes smoked per day following the implementation of a comprehensive tobacco control plan in New York City. *J Urban Health* 2012; **89**(5): 802-8.
7. Smith PH, Rose JS, Mazure CM, Giovino GA, McKee SA. What is the evidence for hardening in the cigarette smoking population? Trends in nicotine dependence in the U.S., 2002-2012. *Drug Alcohol Depend* 2014; **142**: 333-40.
8. Azagba S. Hardcore smoking among continuing smokers in Canada 2004-2012. *Cancer Causes Control* 2015; **26**(1): 57-63.
9. Goodwin RD, Wall MM, Gbedemah M, et al. Trends in cigarette consumption and time to first cigarette on awakening from 2002 to 2015 in the USA: new insights into the ongoing tobacco epidemic. *Tob Control* 2018; **27**(4): 379-84.
10. Lund M, Lund KE, Kvaavik E. Hardcore smokers in Norway 1996-2009. *Nicotine Tob Res* 2011; **13**(11): 1132-9.
