# Supplementary material for: Smokers Increasingly Motivated and Able to Quit as Smoking Prevalence Falls: Umbrella and Systematic Review of Evidence Relevant to the “Hardening Hypothesis,” Considering Transcendence of Manufactured Doubt
Source: Nicotine Tob Res. 2022 Mar 3;24(8):1321–8. doi: 10.1093/ntr/ntac055 (PMC9278822; doi:10.1093/ntr/ntac055)
Supplement: ntac055_suppl_Supplementary_Material_S6 [file ntac055_suppl_supplementary_material_s6.pdf]

Supplementary Material 6: Summary table of author acknowledgements and declarations of interest reproduced from the reviews published to date and primary research studies

**Reviews Published to Date**	
**Warner & Burns (2003)**[1]	Acknowledgements: We thank John Hughes and two anonymous reviewers for helpful comments on earlier drafts of this paper, and Jacqueline Major and Christy Anderson for assistance in preparing the tables and figure.<br>No area stating declaration of competing interest in the article.
**Hughes (2011)**[2]	Acknowledgements: The author thanks David Burns and Kenneth Warner for comments on an early draft of this paper.<br>Role of funding source: The writing of this review was funded by Senior Scientist Award DA-00490 from the National Institute on Drug Abuse. The sponsor had no role in the writing of this report or the decision to submit the report.<br>Conflict of interest: Dr. Hughes is currently employed by The University of Vermont and Fletcher Allen Health Care. Since 1/1/2008, he has received research grants from the National Institute on Health and Pfizer; the latter develops and sells smoking cessation medications. During this time, he has accepted honoraria or consulting fees from several non-profit and for-profit organizations and companies that develop, sell or promote smoking cessation products or services or educate/advocate about smoking cessation: Abbot Pharmaceuticals; Aradigm; American Academy of Addiction Psychiatry; American Psychiatric Association; American Psychiatric Institute for Research and Education; Cambridge Hospital; Dean Foundation; Dartmouth-Hitchcock; DLA Piper; EPI-Q; European Respiratory Society; Evotec; Free and Clear; Glaxo-Smith Kline; Golin Harris; Healthwise; Integrated Communication; Invivodata; Maine Health; McGill University Medical School, McNeil Pharmaceuticals; Novar-tis Pharmaceuticals; Oglivy Health PR, Ottawa Heart Institute, Pfizer Pharmaceuticals; Pinney Associates; Propagate Pharmaceuticals; Reckner Associates; Scientia; University of Arkansas for Medical Sciences; University of California-San Francisco; US National Institutes of Health; Wolters Publishing.
**Hughes (2019)**[3]	Acknowledgements: The author thanks Catherine Peasley-Miklus and the authors who responded to requests about their studies.<br>Declaration of interests: JRH has received consulting and speaking fees from several companies that develop or market pharmacological and behavioral treatments for smoking cessation or harm reduction and from several nonprofit organizations that promote tobacco control. He currently receives consulting fees from Swedish Match and has received fees in the past from Philip Morris and Altria to assist their efforts to develop less-risky tobacco products.
**Review of Primary Research Studies**	
Australian Studies	
**Clare et al (2014)**[4]	Acknowledgements: The National Drug and Alcohol Research Centre at the University of NSW is supported by funding from the Australian Government under the Substance Misuse Prevention and Service Improvements Grants Fund. We would also like to acknowledge The AIHW who conducted the NDSHS surveys on behalf of the Department of Health, and The Australian Social Science Data Archive for providing access to the NDSHS data.<br>Funding: This work was supported by funding received by the National Health and Medical Research Council (grant number APP1021862).<br>Competing interests: None declared.
**Brennan et al (2019)**[5]	Funding: The Victorian Smoking and Health survey was auspiced by Quit Victoria, with funding from VicHealth, the State Government of Victoria and Cancer Council Victoria. The funders had no influence on the decision to submit the paper for publication.<br>Competing interests: None declared.

International Studies	
**Coady et al (2012)**[6]	Funding source: This work was supported by the New York City Department of Health and Mental Hygiene. No outside funding was provided.<br>Declaration of competing interests not provided in article.
**Docherty et al (2014)**[7]	Declaration of interests: G.D., A.M. and L.S. are fully or part-funded by the UK Centre for Tobacco & Alcohol Studies, a UK Clinical Research Collaboration Public Health Research: Centre of Excellence. Funding from the British Heart Foundation, Cancer Research UK, Economic and Social Research Council, Medical Research Council and the National Institute for Health Research under the auspices of the UK Clinical Research Collaboration is gratefully acknowledged. C.G. is funded by a National Health and Medical Research Council (Australia) research fellowship.<br>None of the authors are connected to the tobacco, alcohol, pharmaceutical or gaming industries, or are funded by one of these organizations.
**Kulik & Glantz (2016)**[8]	Funding: This research was funded by National Cancer Institute Grants CA-113710 and CA-060121. The funding agency played no role in the conduct of the research or preparation of the manuscript.<br>Competing interests: None declared.
**Edwards et al (2017)**[9]	Acknowledgments: The authors thank The Health Promotion Agency for supplying the data, producing the survey instruments, managing the data collection process and setting up, and maintaining the data sets. The also thank all the survey participants.<br>Funding: This project was not supported by external funding. It was undertaken as research activity under university and government agency employment.<br>Competing interests: Although we do not consider it a competing interest, for the sake of full transparency we note that one of the authors (RE) has previously undertaken work for health sector agencies working in tobacco control New Zealand Ethics Committee.
**Smith et al (2014)**[10]	Role of funding source: Funding for this study was provided by NIDA, ORWH, and NIMH (P50 DA 03394502, PI: Dr. Sherry McKee; K12 DA031050, PI: Dr. Carolyn Mazure; R21 DA029834, PI: Dr. Jennifer Rose; T32 MH01423539, PI: Dr. Heping Zhang). NIMH and NIDA had no further role in the study design; in the collection, analysis and interpretation of data; in the writing of the report; or in the decision to submit the paper for publication.<br>Conflict of interest: All authors declare that they have no conflicts of interest.
**Azagaba (2015)**[11]	Acknowledgments: This work was supported by a research grant from the Canadian Cancer Society Research Institute (Grant Number 2011-701019).<br>Conflict of interest: None.
**Goodwin et al (2018)**[12]	Funding: This work was supported by grant #2R01 DA20892 (Goodwin) from NIDA.<br>Competing interests: None declared.
**Lund et al (2011)**[13]	Funding: The present study was supported by the Research Program on Public Health (FOLKEHELSE) of the Research Council of Norway, project no.190443 “Tobacco and the social inequality gap,” and from Norwegian Institute for Alcohol and Drug Research.<br>Acknowledgements: Thanks to the Norwegian Directorate for Health for initiating the survey, Statistics Norway for collecting them, and Norwegian Social Science Data Service for making data available. Neither of the institutes above is responsible for the analysis or interpretations in this article.<br>Declaration of Interests – none declared.

References

1. Warner KE, Burns DM. Hardening and the hard-core smoker: concepts, evidence, and implications. *Nicotine Tob Res* 2003; **5**(1): 37-48.
2. Hughes JR. The hardening hypothesis: is the ability to quit decreasing due to increasing nicotine dependence? A review and commentary. *Drug Alcohol Depend* 2011; **117**(2-3): 111-7.
3. Hughes JR. An Update on Hardening: A Qualitative Review. *Nicotine Tob Res* 2019; **22**(6): 867-71.
4. Clare P, Bradford D, Courtney RJ, Martire K, Mattick RP. The relationship between socioeconomic status and 'hardcore' smoking over time--greater accumulation of hardened smokers in low-SES than high-SES smokers. *Tob Control* 2014; **23**(e2): e133-8.
5. Brennan E, Greenhalgh EM, Durkin SJ, Scollo MM, Hayes L, Wakefield MA. Hardening or softening? An observational study of changes to the prevalence of hardening indicators in Victoria, Australia, 2001-2016. *Tob Control* 2019; **29**: 252-7.
6. Coady MH, Jasek J, Davis K, Kerker B, Kilgore EA, Perl SB. Changes in smoking prevalence and number of cigarettes smoked per day following the implementation of a comprehensive tobacco control plan in New York City. *J Urban Health* 2012; **89**(5): 802-8.
7. Docherty G, McNeill A, Gartner C, Szatkowski L. Did hardening occur among smokers in England from 2000 to 2010? *Addiction* 2014; **109**(1): 147-54.
8. Kulik MC, Glantz SA. The smoking population in the USA and EU is softening not hardening. *Tob Control* 2016; **25**(4): 470-5.
9. Edwards R, Tu D, Newcombe R, Holland K, Walton D. Achieving the tobacco endgame: evidence on the hardening hypothesis from repeated cross-sectional studies in New Zealand 2008-2014. *Tob Control* 2017; **26**(4): 399-405.
10. Smith PH, Rose JS, Mazure CM, Giovino GA, McKee SA. What is the evidence for hardening in the cigarette smoking population? Trends in nicotine dependence in the U.S., 2002-2012. *Drug Alcohol Depend* 2014; **142**: 333-40.
11. Azagba S. Hardcore smoking among continuing smokers in Canada 2004-2012. *Cancer Causes Control* 2015; **26**(1): 57-63.
12. Goodwin RD, Wall MM, Gbedemah M, et al. Trends in cigarette consumption and time to first cigarette on awakening from 2002 to 2015 in the USA: new insights into the ongoing tobacco epidemic. *Tob Control* 2018; **27**(4): 379-84.
13. Lund M, Lund KE, Kvaavik E. Hardcore smokers in Norway 1996-2009. *Nicotine Tob Res* 2011; **13**(11): 1132-9.
